# Metabolic potential of the organic-solvent tolerant *Pseudomonas putida* DOT-T1E deduced from its annotated genome

**DOI:** 10.1111/1751-7915.12061

**Published:** 2013-07-01

**Authors:** Zulema Udaondo, Lazaro Molina, Craig Daniels, Manuel J Gómez, María A Molina-Henares, Miguel A Matilla, Amalia Roca, Matilde Fernández, Estrella Duque, Ana Segura, Juan Luis Ramos

**Affiliations:** 1 Estación Experimental del Zadín-CSICProfesor Albareda 1, 18008, Granada, Spain; 2 CIDERTA, LICAH, Parque Huelva EmpresarialIntersección 80 de la Autovía A-49, 21007, Huelva, Spain; 3 Department of Anesthesia, University of Toronto150 College Street, Room 121 FitzGerald Building, Toronto, Ontario, M5S 3E2, Canada; 4 INTA-CSICCarretera de Ajalvir km 4, 28850, Torrejón de Ardoz (Madrid), Spain; 5 Bio-Iliberis R&DC/ Capileira 7, 18210 Peligros, Granada, Spain

## Abstract

*Pseudomonas putida* DOT-T1E is an organic solvent tolerant strain capable of degrading aromatic hydrocarbons. Here we report the DOT-T1E genomic sequence (6 394 153 bp) and its metabolic atlas based on the classification of enzyme activities. The genome encodes for at least 1751 enzymatic reactions that account for the known pattern of C, N, P and S utilization by this strain. Based on the potential of this strain to thrive in the presence of organic solvents and the subclasses of enzymes encoded in the genome, its metabolic map can be drawn and a number of potential biotransformation reactions can be deduced. This information may prove useful for adapting desired reactions to create value-added products. This bioengineering potential may be realized via direct transformation of substrates, or may require genetic engineering to block an existing pathway, or to re-organize operons and genes, as well as possibly requiring the recruitment of enzymes from other sources to achieve the desired transformation.

**Funding Information** Work in our laboratory was supported by Fondo Social Europeo and Fondos FEDER from the European Union, through several projects (BIO2010-17227, Consolider-Ingenio CSD2007-00005, Excelencia 2007 CVI-3010, Excelencia 2011 CVI-7391 and EXPLORA BIO2011-12776-E).

Bacteria of the genus *Pseudomonas* are motile Gram-negative bacteria characterized by high metabolic versatility, and aerobic respiration, although a few strains of different species are able to use nitrate as a final electron acceptor (Palleroni, 2010). Pseudomonads are ubiquitous soil and water microorganisms that colonize many different environments and, consequently, have diverse lifestyles. Strains of the species *Pseudomonas putida* are frequently soil inhabitants and are important in organic matter recycling in nature; they have a high bioremediation potential because they often carry genes to deal with natural and xenobiotic chemicals (Nelson *et al*., 2002; Caballero *et al*., 2005; van Dillewijn *et al*., 2007; Arias *et al*., 2008; Segura *et al*., 2009a,2009b). The key to the ubiquitous distribution of these bacteria is not only their metabolic potential, but also the arsenal of regulatory genes that allow them to adapt to changes in the environment (Sashidhar and Podile, 1998; Wu *et al*., 2011). A few *P. putida* strains, namely, S12, Idaho and DOT-T1E (Weber *et al*., 1993; Ramos *et al*., 1995; Pinkart *et al*., 1996), are able to thrive in the presence of toxic solvents (e.g. decanol, octanol, toluene, styrene), and these strains are considered extremophile microorganisms with great potential in bioremediation and in biocatalysis in biphasic systems (Ramos *et al*., 1995; Isken and de Bont, 1996; Molina *et al*., 2011; Ramos *et al*., 2011; Tao *et al*., 2011; Udaondo *et al*., 2012). Organic solvents are toxic to most microorganisms because they dissolve in the cell membranes, disorganize their structures and impair vital functions such as respiration, and the collapse in energy generation lead to cell death (Sikkema *et al*., 1995; Ramos *et al*., [Bibr b48],[Bibr b51]). Solvent tolerance in *P. putida* DOT-T1E is a multifactorial trait that involves chromosomal and plasmid encoded functions (Ramos *et al*., 2002; Segura *et al*., 2003; 2009b; Rodríguez-Herva *et al*., 2007; García *et al*., 2006; Molina *et al*., 2011). The first barrier to solvents involves a reduction in the permeability of the cell membrane via a fast *cis* to *trans* isomerization of unsaturated fatty acids followed by a slower modification of phospholipid head groups (Keweloh and Heipieper, 1996; Junker and Ramos, 1999; Heipieper *et al*., [Bibr b19],[Bibr b20]; Bernal *et al*., 2007; Pini *et al*., 2009). However, this reduction in permeability does not prevent entry of the solvents, which results in unfolding of proteins and the consequential function of a number of chaperones (Segura *et al*., 2003; Domínguez-Cuevas *et al*., 2006; Volkers *et al*., 2006). The main mechanism underlying solvent tolerance lies in the action of RND (resistance-nodulation-cell division) efflux pumps encoded on the host chromosome and on the pGRT1 plasmid (Kieboom *et al*., 1998; Kim *et al*., 1998; Ramos *et al*., 1998; Mosqueda and Ramos, 2000; Rojas *et al*., 2001; Rodríguez-Herva *et al*., 2007; Godoy *et al*., 2010; Udaondo *et al*., 2012). Resistance to solvents is also modulated by the action of chromosomally encoded ABC efflux transporters that use energy to remove solvents from the cells to the outer medium (Kim *et al*., 1998; García *et al*., 2006).

Here, we present, the genome of the solvent tolerant *P. putida* DOT-T1E strain obtained using the *454* technology. This microorganism uses a wide range of carbon, nitrogen, sulfur and phosphorous sources due to its wide metabolic potential. In addition we summarize previous knowledge on the biotransformation potential of this strain and how the properties and genomic information can be used to design new biotechnological processes.

## Genome sequencing, assembly, annotation and bioinformatic analysis

**Figure 1:**
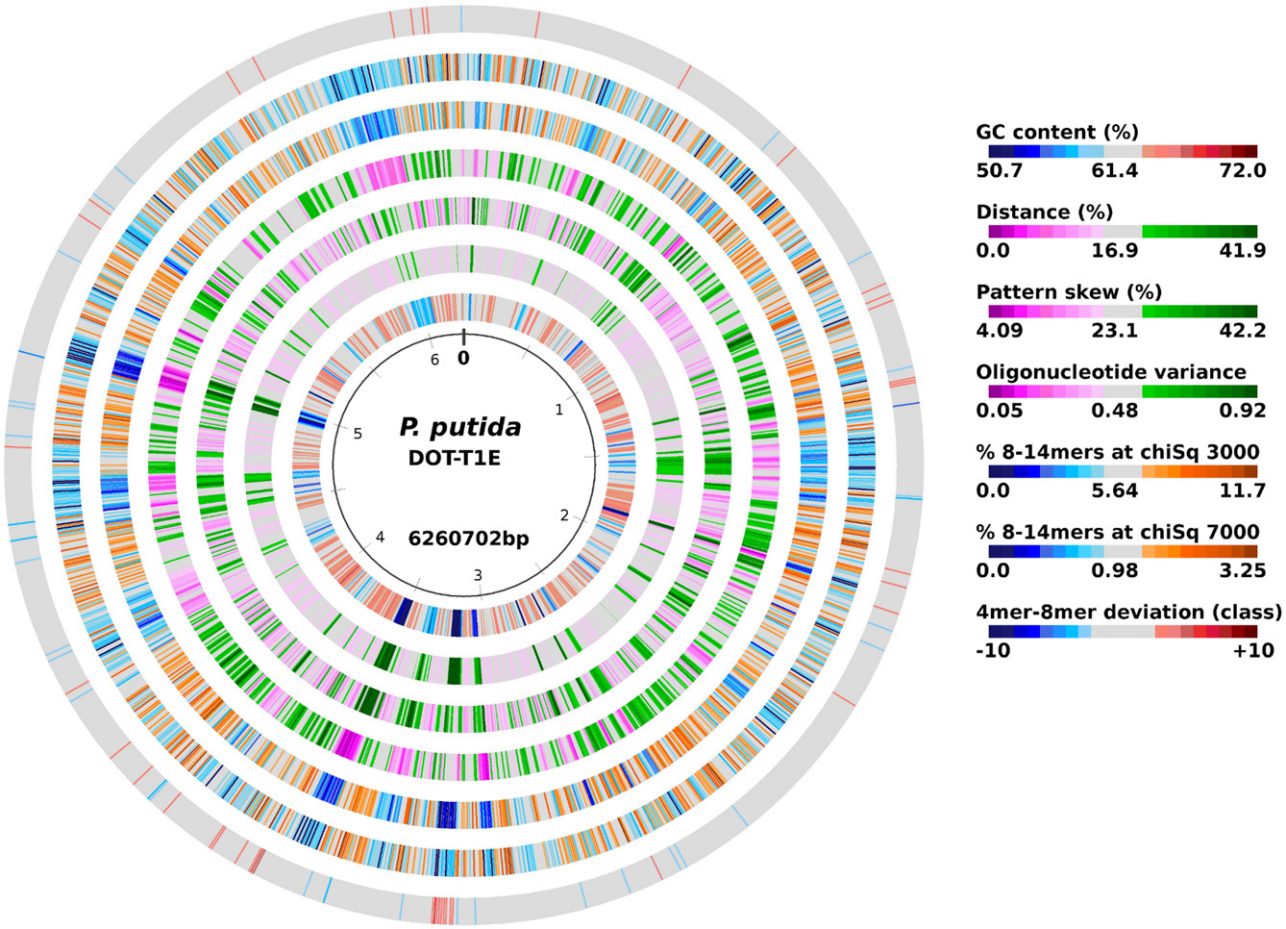
Circular genome of *Pseudomonas putida* DOT-T1E. G+C content and the three tetranucleotide parameters are plotted on the innermost four rings. Distance (second innermost circle) is the distance between global and local sliding window tetranucleotide patterns. Pattern skew (third inner most circle) is the distance between tetranucleotide rankings on direct and reverse strands. Oligonucleotide variance (fourth inner most circle) is the numerical variance of oligomers, where a lower value indicates tetramer usage and is more highly restricted (for example in repeat regions) (Klockgether *et al*., 2011). The third and second outermost circles show the frequency of distribution of overrepresented (χ^2^ > 3000) and highly overrepresented (χ^2^ > 7000) 8–14 mers in the genome of *P. putida* DOT-T1E. The outermost ring visualizes differences between tetranucleotide usage and the frequency of the overrepresented longer oligomers. Figures were created with JcircleGraph (Davenport *et al*., 2009).

The complete genome sequence of *P. putida* DOT-T1E (GenBank accesion number CP003734) was determined by applying a strategy combining whole-genome-shotgun *454*-pyrosequencing on the genome sequencer FLX platform (20× coverage with 305 contigs, 257 354 nt biggest contig) and Sanger sequencing of PCR amplicons covering gaps between contigs. In addition, after a first round of annotation, regions of lower quality as well as regions with putative frameshifts were resequenced from PCR amplification of the dubious regions and the complete genome sequence was established. The genome of *P. putida* DOT-T1E has two circular replicons; a single chromosome of 6 260 702 base pairs with a G+C content of 61% (Fig. 1) and another of 133 451 base pairs corresponding to a plasmid previously named pGRT1 (Molina *et al*., 2011; Genebank HM626202.1, NCBI Reference Sequence: NC_015855.1).

**Figure 2:**
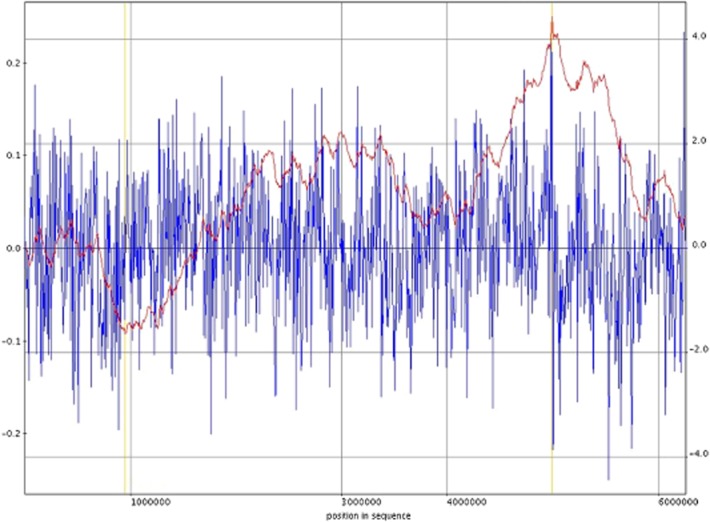
*Pseudomonas putida* DOT-T1E chromosome GC Skew analysis. Gen Skew is defined as the normalized excess of G over C in a given sequence. It is given by (G−C)/(G+C), and it is calculated with a sliding window of 1000 nucleotides along the genome. It is represented in blue. The cumulative GC-skew is the sum of the values of neighbouring sliding windows from an arbitrary start to a given point in the sequence and it is represented in red. GC-skew is positive in the leading strand and negative in the lagging strand.

Using a combination of the Glimmer 3.03 software (Salzberg *et al*., 1998; Delcher *et al*., 1999), blast analysis and manual curation a total of 5803 ORFs were predicted and annotated in the chromosome, of which 170 have no significant homology (at E-value of < 10^−5^) to any ORF present among the sequenced *Pseudomonas* genomes. The analysis of ORFs of pGRT1 was performed previously and revealed it encodes for 126 proteins (Molina *et al*., 2011). In total the genome of DOT-T1E encodes 5721 proteins and 82 RNAs of which 58 corresponded to tRNAs. We analysed the GC skews of the T1E chromosome, which is defined as the value of [G−C]/[G+C] where G and C represent the local base frequencies of G and C respectively. In prokaryotic genomes the GC skew tends to have a positive value on the leading strand of DNA synthesis and a negative value on the lagging strand, resulting in polarity changes at the origin and terminus of replication (Bentley and Parkhill, 2004). The putative position of the replicative terminus is therefore operationally defined as the peak of the cumulative GC skew and it typically resides opposite to the origin of replication in bacterial genomes (Fig. 2) (Bentley and Parkhill, 2004). For T1E, the peak GC skew was indeed mapped opposite the replication origin (at 49.2%) of the genome. We also found that the *oriC* site locates between the *rpmH* and *dnaA* genes and contains two identical boxes (5′-TTATCCACA-3′) with the first T of the first box corresponding to position 991824 while the last A of second box is 991893. Gene names were taken from Best Blast Hit when available, and gene products were classified into COG category (Tatusov *et al*., 2003), Pfam, Prk and Smart families with RPSBlast. Putative ribosomal binding sites and tRNA genes were identified with Rfam (Griffiths-Jones *et al*., 2010) and tRNAscan-SE (Lowe and Eddy, 1997). Manual validation and visualization of the entire metabolic potential of DOT-T1E was performed using the Pathway Tools Program v.16.0 (http://bioinformatics.ai.sri.com/ptools/) (Letunic *et al*., 2008; Karp *et al*., 2010), which allows graphic visualization of the *P. putida* annotations. Analyses were performed using an Intel(R) Core (TM)i 7-2600 CPU 3.40 GHz with 8 Gb of RAM memory running a linux Ubuntu 11.04 operating system. Gene products were analysed, compared and assigned to metabolic pathways according to the MetaCyc scheme (Caspi *et al*., 2008), and published research articles. The cut-off criteria for identifying orthologous proteins were compiled by protein–protein pairwise analysis and reciprocal tBLASTN analysis to identify mutual best hits as potential orthologues. The functional annotations of DOT-T1E genes were corrected for consistency with their counterparts in *P. putida* KT2440 and *P. putida* F1. The coordinates of numerous genes were adjusted according to the criteria of full-length alignment, plausible ribosome binding sites, and minimal overlap between genes on opposite DNA strands. Figure 1 shows the Genome Atlas of *P. putida* (Ussery *et al*., 2009).

We analysed the genome to identify potential genomic islands using three different algorithms based on: (i) lack of continuity in the genome, (ii) alignment to other *P. putida* strains and (iii) G+C content and codon usage. This yielded four island regions, 1 504 914–1 553 486; 3 046 659–3 066 609; 4 526 081–4 539 056 and 4 945 609–4 985 959. Most ORFs in these four islands encode hypothetical proteins of unknown function. ORFs in islands 1 and 4 exhibit no homology with any other known sequence, although significant homology was found with transposases. ORFs in island 3 and 2 are conserved in *P. putida* ND6, a strain that degrades naphthalene (Li *et al*., 2012).

## Metabolic potential

**Figure 3:**
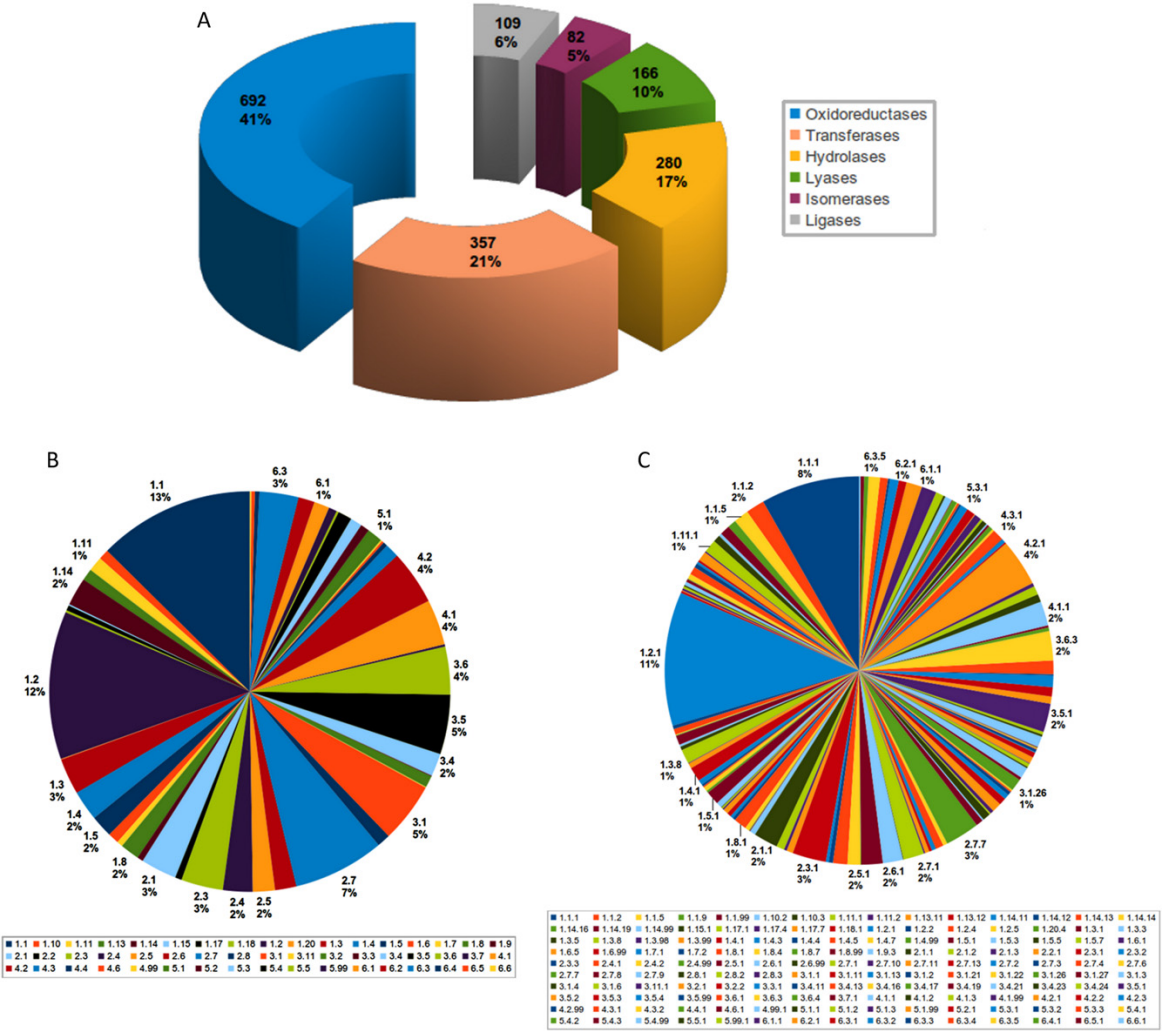
Distribution of enzyme activities of *P. putida* DOT-T1E classified according to the EC nomenclature. (A) EC X; (B) EC XX; and (C) EC XXX. Colour code for classes and subclasses by numbers are indicated. For full details of the EC classification the reader is referred to http://www.chem.qmul.ac.uk/iubmb/enzyme/.

As indicated above analysis of the entire metabolic potential of DOT-T1E was performed using the Pathway Tools Program v.16.0 (http://bioinformatics.ai.sri.com/ptools/) (Karp *et al*., 2002; Letunic *et al*., 2008). In the genome of *P. putida* DOT-T1E we identified up to 1751 enzymatic reactions performed by approximately 1686 enzymes with 1268 unique potential substrates. A numerical classification for the enzymes based on the chemical reactions they carried out according to the Enzyme Commission number (EC number) was elaborated in order to understand the metabolic potential of this strain. According to EC nomenclature (Bairoch, 2000), oxidoreductases (EC 1) were the most abundant enzymes, representing 41% of the total (Fig. 3A). Enzymes belonging to EC classes 2 (transferases), EC classes 3 (hydrolases) and 4 (lyases) represented 21%, 17% and 10% of all enzymes respectively, while isomerases (EC 5) and ligases (EC 6) were the least abundant, with 5% and 6% of total enzymes respectively. This is consistent with the scenario of a high metabolic versatility described for Pseudomonads (Daniels *et al*., 2010; Palleroni, 2010).

The second level of EC nomenclature (EC X.X) includes a total of 65 subclasses, of which 51 are present in *P. putida* DOT-T1E (Fig. 3B). As expected, from the high number of oxidoreductases, two subclasses of this group were among the most abundant with enzymes that use the CH-OH group as donor (EC 1.1) and those using aldehyde as donors (EC 1.2) representing nearly 12% of the total for each group. A striking observation was the presence of certain abundant enzyme classes, such as for example phosphotransferases (EC 2.7, 7% of total); and a series of hydrolases acting on carbon-nitrogen bonds (EC 3.5, 5% of total), or acting on ester bonds and anhydrides (EC 3.1; about 5% of total). Figure 3B presents the enzymes of DOT-T1E grouped based on their subclasses. We further classified the enzymes identified in functional subclasses according to the EC X.X.X nomenclature to focus on the potential donors and acceptors in the case of oxidoreductase enzymes or potential groups of substrates in other enzymes (Fig. 3C). Among a total number of 269 subclasses in the third level of EC nomenclature (EC X.X.X), 150 were present in *P. putida* DOT-T1E. Oxidoreductases using aldehydes as donor groups with NAD^+^ or NADP^+^ as acceptor (EC 1.2.1) were the most abundant (11% of the total), also numerically important were the carbon-oxygen lyases (EC 4.2.1, 4% of total), nucleotidyl phosphotransferases (EC 2.7.7, 3% of total) and acyltransferases (EC 2.3.1, 3% of total).

The enzyme data sets were additionally used to analyse potential substrates and to generate a complete list of enzyme distribution per functional category EC X.X.X.X, the data for which is shown in Table S1. Using the Pathway Tool platform, the set of phenomics assays previously described by our group (Daniels *et al*., 2010), and the EC X.X.X classification allowed us to explain the pattern of growth of strain DOT-T1E with 65 different carbon sources, 60 nitrogen sources, and 15 sulfur sources used as nutrients (Table S2). In total 425 pathways for metabolism of different compounds were delineated. This analysis confirms the limited ability of *P. putida* to use sugars as a C source, which is restricted to glucose, gluconate and fructose. DOT-T1E has a complete Entner–Doudoroff route for utilization of glucose and other hexoses, but lacks the 6-phosphofructokinase of the glycolytic pathway, in agreement with the genome analysis of others Pseudomonads (del Castillo *et al*., 2007). A large number of sugars were found to not be metabolized by T1E including xylulose, xylose, ribulose, lyxose, mannose, sorbose, d-mannose, alginate, rhamnose, rhamnofuranose, galactose, lactose, epimelibiose, raffinose, sucrose, stachyose, manninotriose, melibiose, tagatose, starch and cello-oligosaccharides, to cite some, in agreement with the lack of genes for the metabolism of these chemicals after the genome analysis of this strain. The results also confirmed the ability of *P. putida* to use as a C source organic acids (such as acetic, citric, glutaric, quinic, lactic and succinic among others), certain *L-*amino acids (Ala, Arg, Asn, Glu, His, Ile, Lys, Pro, Tyr and Val), and various amino organic compounds. (See Figs S1–S4 for examples of catabolic pathways for sugars, amino acids, organic acids and aromatic compounds catabolism.)

Strain T1E harbours genes for a limited number of central pathways for metabolism of aromatic compounds and numerous peripheral pathways for funnelling of aromatic compounds to these central pathways. As in other Pseudomonads one of the strategies exploited by this microbe for the degradation of different aromatic compounds is to modify their diverse structures to common dihydroxylated intermediates (Dagley, 1971); another strategy is to generate acyl-CoA derivatives such as phenylacetyl-CoA (Fernández *et al*., 2006). Regarding peripheral pathways the *P. putida* DOT-T1E genome analysis has revealed determinants for putative enzymes able to transform a variety of aromatic compounds. The DOT-T1E strain is able to use aromatic hydrocarbons such as toluene, ethylbenzene, benzene and propylbenzene to cite some (Mosqueda *et al*., 1999). The strain also uses aromatic alcohols such as conyferyl- and coumaryl-alcohols and their aldehydes; a range of aromatic acids such as ferulate, vanillate, *p*-coumarate, *p*-hydroxybenzoate, *p*-hydroxyphenylpyruvate, phenylpyruvate, salicylate, gallate and benzoate (see Fig. S4). These chemicals are channelled to central catabolic pathways. Upon oxidation of these chemicals they are metabolized through one of the three central pathways for dihydroxylated aromatic compounds present in this strain. The β-ketoadipate pathway is a convergent pathway for aromatic compound degradation widely distributed in soil bacteria. This pathway consists of a catechol branch (*cat*) and protocatechuate branch (*pca*). The *pca* genes in *P. putida* DOT-T1E are arranged in three operons [*pcaRKFTBDC* (T1E_0230 through T1E_0238), *pcaGH* (T1E_0829 and T1E_830), *pcaJI* (T1E_2058 and T1E_2059)], as is also the case in other *P. putida* and *P. syringae* strains (Fig. S5).

The *cat* genes encode the proteins responsible for catechol degradation and are organized in two clusters [*catRBCA* (T1E_5502 through T1E_5505) and *catBCA* (T1E_1744 through T1E_ 1746)] (Fig. S6), maintaining the gene order found in others *P. putida* strains and also in *P. aeruginosa*. The identity of the *catBC* and *A* genes in both clusters is in the range of 79–82%. In addition, we should mention that two other *catA* genes were found, one of them with a high degree of similarity to the KT2440 *catA2* gene, which corresponded to ORF T1E_1057, that is adjacent to the *benRABCDK* genes (T1E_1055 to T1E_1064) for benzoate degradation; while the other *catA* allele corresponded to ORF T1E_5511. It should be noted that this allele is within a cluster of genes that are transcribed in the same direction and which encode genes for salycilate metabolism (T1E_5510 through T1E_5513).

The genes involved in phenylacetate degradation were also identified in *P. putida* DOT-T1E. There are 16 genes encoding for phenylacetate degradation organized in a cluster (ORFs T1E_5587 to T1E_5603) and within the cluster a series of potential operons were identified, i.e. the *paaGHIJK* genes (T1E_5590 through T1E_5594) that encode the ring-hydroxylating oxygenase enzyme, the *paaABCDE* genes that encode the β-oxidation enzymes, a potential phenylacetate transport system (*paaLM*) and the regulatory system made of *paaXY*, that correspond to T1E_5587 and T1E_5588 respectively.

Homologous genes for degradation of homogentisate are also present in strain DOT-T1E. Homogentisate is catabolized by a central catabolic pathway that involves three enzymes, homogentisate dioxygenase (T1E_1557), a newly identified putative maleylacetoacetate isomerase (T1E_1555) and fumarylacetoacetate hydrolase (T1E_1558). In this pathway homogentisate is funnelled to yield fumarate and acetoacetate. A search for *hpa* and *gtd* genes that encode genes belonging to the homoprotocatechuate and gentisate pathways yielded no results from the DOT-T1E genome, which suggests the absence of a *meta* ring-cleavage pathway for the degradation of homoprotocatechuate and gentisate.

**Figure 4:**
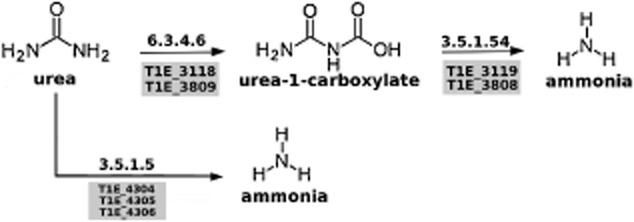
Pathway for utilization of urea as an N source by *P. putida*. The genes that encoded the enzymes of these two pathways were identified based on blast analysis and comparison to proteins that carry out the indicated reactions.

Pseudomonads strains are able to use a range of inorganic nitrogen sources. In this regard three predicted transporters involved in the uptake of ammonium were identified. T1E incorporates ammonium into C skeletons using mainly the ATP-dependent activity of glutamine synthetase (GS) followed by the action of glutamate synthase (GOGAT). The genome of T1E encodes four GS (T1E_0118, 1260, 2050 and 4444) and four GOGAT enzymes (T1E_1644, 2053, 2506 and 3293). Strain T1E can use nitrate as an N source, which is reduced to ammonium using an assimilatory nitrate reductase (EC: 1.7.99.4) encoded by the T1E_4793 gene, that is in a cluster with *nirB* and *nirD* which encode an assimilatory nitrite reductase (EC1.7.1.4). (The ORFs encoding these proteins correspond to T1E_4793 through T1E_4795.) The strain also has the complement of genes for utilization of urea either through direct conversion to ammonia (T1E_4304 through T1E_4306, *ureABC*) or via conversion first to urea-1-carboxylate (T1E_3118 through and 3809) and then conversion to ammonia (T1E_3119 and T1E_3808) (Fig. 4).

Details for the utilization of *D*- and *L*-amino acids as N sources were published by Daniels and colleagues (2010). It was found that the wild-type DOT-T1E strain was able to use a number of either *D*- or *L*-amino acids (i.e. *D*-ornithine, *D*-alanine, *D*-arginine, *D*-asparagine, *D*-lysine and *D*-valine), dipeptides, ethanolamine, and adenine as an N source (Daniels *et al*., 2010). It is of interest to highlight that this strain can use several *D*-amino acids for which racemases are needed. We have found that the genome of DOT-T1E encodes at least five broad-substrate racemases (T1E_2780, TIE_3429, TIE_1731, TIE_0166, TIE_4880) that can convert *D*-amino acids into *L*-amino acids which upon transamination allow the catabolism of these compounds to provide nitrogen for growth (Daniels *et al*., 2010). Eight aminopeptidases (TIE_3567, TIE_2564, TIE_4792, TIE_1957, TIE_2243, TIE_3241, TIE_3898, TIE_0833) also allows this bacterium to utilize a number of dipeptides and tripeptides as C- and N- sources, in agreement with the saprophytic character of strains of this species (Daniels *et al*., 2010).

Strain T1E has a number of genes that may encode enzymes/transporters needed for the acquisition of inorganic phosphate, namely: (i) two low-affinity Pit type transporters (T1E_0227 and T1E_0045), (ii) two putative ABC-type inorganic phosphate high-affinity transporter (T1E_2661 through 2663 and T1E_3987 through 3989) and (iii) a PstS type (T1E_2660) high-affinity transporter system regulated by the *phoBR* (T1E_3994 and 3993) response regulator system. This strain uses organic phosphate ester compounds under phosphorous-limiting conditions (Daniels *et al*., 2010). T1E also use organic phosphonates that are transported by a high-affinity ABC transport system consisting of the *phnD*, *phnE* and *phnC* gene products (T1E_4609 through 4612).

Members of the pseudomonadaceae have been reported to play a key role in mineralization of carbon bound sulfur in rhizosphere soils. Organic sulfur in soils is comprised mostly of sulfonates and sulfate esters; hence, many soil bacteria carry genes that encode enzymes for utilization of alkanesulfonates. Metabolism of these compounds is achieved through the action of the Ssu enzymes, which are encoded by a set of genes that form an operon, namely, *ssuA* through *F* (T1E_2976 through 2982). This organization is similar to that in other *Pseudomonas* (Kahnert and Kertesz, 2000). The strain DOT-T1E is also endowed with at least one putative arylsulfatase (T1E_5507) which may explain the ability of the strain to use aromatic sulfate esters (Daniels *et al*., 2010). The DOT-T1E strain is also endowed with four genes that may encode the enzymes required to make sulfur available from methionine (T1E_0568, T1E_2981, T1E_4829 and T1E_4830), which is released as sulfite (Fig. S7). The set of reactions is initiated by MdeA as in other pseudomonads and the pathway is depicted in Fig. S7.

A relevant characteristic of DOT-T1E is its capability to grow on minimal medium without the need of vitamins or other cofactors. We found 165 genes encoding enzymatic reactions mediating the biosynthesis of a number of cofactors, i.e. nicotinate, nicotinamide, vitamin B6, riboflavin, ubiquinone, porphyrin, biotin, thiamine, folate, pantothenate and CoA which amounts for 74 distinct biosynthetic pathways. This is consistent with a metabolism in which different enzymes have been described to use these molecules as cofactors.

Based on phenotypic analysis using the BIOSCREEN growth test system described by Daniels and colleagues (2010), it was shown that *P. putida* T1E tolerated various heavy metals. Based on the strain's genome sequence, 64 genes were identified that encode proteins putatively involved in heavy metal resistance and homeostasis (Table 1). The majority of the *P. putida* T1E heavy metal resistance genes are found spread throughout the genome, and they are conserved among all sequenced *P. putida* strains.

**Table 1:** Proteins found in *P. putida* DOT-T1E that are associated with metal resistance and homeostasis

Gene location	Protein name	Metal	Family/domain	Predicted role	Definition	E-value
T1E_0296	CzcS2	Me^2^	TC reg	Sensor protein	Hypothetical protein	0.0
T1E_0297	CzcR2	Me^2^	TC reg	Response regulator	DNA-binding response regulator CzrR	e-126
T1E_0503	TPMT	Te, Se	TPMT	Te and Se Se/Te detoxification	Thiopurine *S*-methyltransferase	e-122
T1E_0621	ZnuA2	Zn/Mn(?)	PBD	Zn/Mn(?) uptake	Periplasmic solute-binding protein	e-167
T1E_0622	ZnuC2	Zn/Mn(?)	ATP-binding protein	Zn/Mn(?) uptake	Cation ABC transporter, AP-binding protein	e-122
T1E_0658	Fur	Fe	Fur	Fe regulation	Ferric uptake regulator, Fur family	7e-073
T1E_0727	CopA	Cu	HMA	Copper exporting ATPase	Heavy metal translocating P-type ATPase	0.0
T1E_1144	ArsR3	As, Sb	ArsR	Transcriptional regulator	ArsR family transcriptional regulator	5e-152
T1E_1232	CopS2	Cu	TC reg	Response regulator	Heavy metal sensor signal transduction histidine kinase	0.0
T1E_1233	CopR2	Cu	TC reg	Sensor protein	Two-component heavy metal response transcriptional regulator	e-122
T1E_1234	T1E_1234	Cu	Cupredoxine	Copper homeostasis	Plastocyanin/azurin family copper-binding protein	4e-072
T1E_1474	ModC	Mo	ATP-binding protein	Mo uptake	Molybdate ABC transporter ATPase	0.0
T1E_1475	ModB	Mo	I M pore	Mo uptake	Molybdate ABC transporter inner membrane protein	e-128
T1E_1476	ModA	Mo	PBD	Mo uptake	Molybdenum ABC transporter periplasmic molybdate-binding protein	e-136
T1E_1824	NikE	Ni	ATP-binding protein	Ni uptake	Nickel transporter ATP-binding protein NikE	3e-041
T1E_1827	NikB	Ni	I M pore	Ni uptake	Nickel transporter permease NikB	7e-050
T1E_2011	CumA	Cu	Cu oxidase	Copper homeostasis	Multicopper oxidase	0.0
T1E_2070	NikC	Ni	I M pore	Ni uptake	Nickel transporter permease NikC	4e-059
T1E_2193	ModR	Mo	modE	Mo uptake regulation	Mode family transcriptional regulator	e-127
T1E_2274	T1E_2274	Cu	(MFS) transporters	Copper homeostasis	Bcr/CflA family multidrug resistance transporter	0.0
T1E_2279	T1E_2279	Co/Zn/Cd	OEP	Cobalt-zinc-cadmium resistance	Heavy metal RND efflux outer membrane protein, CzcC family	0.0
T1E_2577	ZnuB2	Zn/Mn(?)	I M pore	Zn/Mn uptake	hypothetical protein	e-160
T1E_2719	ArsH2	As, Sb	ArsH	Arsenical resistant	ArsH protein	e-132
T1E_2720	ArsC2	As, Sb	ArsC	As(V) reduction	Arsenate reductase	8e-086
T1E_2721	ArsB2	As, Sb	ArsB	As(III), Sb(III) efflux	Arsenite efflux transporter	0.0
T1E_2722	ArsR2	As, Sb	ArsR	Transcriptional repressor	Arsenic resistance transcriptional regulator	8e-063
T1E_2794	NikA	Ni	PBR	Ni uptake	Nickel ABC transporter, periplasmic nickel-binding protein	1e-039
T1E_2808	CzcD	Me^2^	CDF	Transport and regulation	CDF family cobalt/cadmium/zinc transporter	e-166
T1E_2811	CzcR1	Me^2^	TC reg	Response regulator	DNA-binding response regulator CzrR	e-124
T1E_2812	CzcS1	Me^2^	TC reg	Sensor kinase	Sensor histidine kinase	0.0
T1E_2820	CadA1	Zn/Cd	P-type ATPase	Me2+ efflux	Heavy metal translocating P-type ATPase	0.0
T1E_2933	TetR	Drug(?)	TetR	Transcriptional regulator	TetR family transcriptional regulator	e-117
T1E_3354	ChrA	Cr	ChrA	Chromate efflux	Chromate transporter	0.0
T1E_3756	PacR(CueR)	Cu/Ag	MerR	Transcriptional regulaor	MerR family transcriptional regulaor	1e-075
T1E_3757	PacS	Cu	P-type	Cooper uptake	Heavy metal translocating P-type ATPase	0.0
T1E_3759	PacZ(CopZ)	Cu	HMA	Activator	heavy metal transport/detoxification protein	7e-031
T1E_3760		Cu		Cooper homeostasis	Multidrug resistance transporter, Bcr/CflA family	1e-163
T1E_4452	MfpII	Me^2^/drug	RND MFP/HlyD	Me2+/drug efflux	efflux transporter, RND family, MFP subunit	0.0
T1E_4453	MfpI	Me^2^/drug	RND MFP/HlyD	Me2/drug efflux	RND efflux transporter	0.0
T1E_4454	CzcA4	Me^2^/drug	RND	Me2/drug efflux	Acriflavin resistance protein	0.0
T1E_4488	CadR	Zn/Cd	MerR	Cd, Zn efflux	MerR family transcriptional regulator	8e-082
T1E_4489	CadA2	Cd/Zn	P-type ATPase	Cd, Zn efflux	Heavy metal translocating P-type ATPase	0.0
T1E_4513	CopA1	Cu	MultiCU oxidases	Cu chelation	Copper resistance protein A	0.0
T1E_4672	ZnuC1	Zn	ATP-binding protein	Zn uptake	Zinc ABC transporter ATP-binding protein	e-147
T1E_4694	CusA	Me^2^	RND	Me2 efflux	CzcA family cobalt/zinc/cadmium efflux transporter permease	0.0
T1E_4695	CusB	Me^2^	RND MFP/HlyD	Me2+ efflux	CzcB family cobalt/zinc/cadmium efflux transporter membrane fusion protein	0.0
T1E_4696	CusC	Me^2^	OEP	Me2+ efflux	CzcC family cobalt/zinc/cadmium efflux transporter outer membrane protein	0.0
T1E_4697	PorD		Porin	Channel basic amino acids	Porin, putative	0.0
T1E_4698	CzcR3	Me^2^	TC reg	Response regulator	DNA-binding heavy metal response regulator, putative	e-127
T1E_4760	ZnuA1	Zn	PBD	Zn uptake	Periplasmic solute-binding protein	e-171
T1E_4761	Zur	Zn	Fur	Regulator	FUR family transcriptional regulator	2e-074
T1E_4763	ZnuB1	Zn	I M pore	Zn uptake	Hypothetical protein	e-138
T1E_4936		Cu	CBS	Copper homeostasis	CBS domain containing protein	1e-130
T1E_4939	ArsR1	As, Sb	ArsR	Transcriptional regulator	ArsR family transcriptional regulator	5e-048
T1E_4996	ArsC3	As, Sb		Arsenate reductase	Arsenate reductase	1e-060
T1E_5088	CzcA5	Me^2^/drug	RND	Me2+/drug efflux	Acriflavin resistance protein	0.0
T1E_5089	Mtrc2	Me^2^/drug	RND MFP/HlyD	Me2+/drug efflux	Efflux transporter, RD family, MFP subunit	0.0
T1E_5270	CzcA2	Me^2^	RND	Cation efflux	Cobalt-zinc-cadmium resistance protein CzcA	0.0
T1E_5271	CzcB2	Me^2^	RND MFP/HlyD	Cation efflux	Cobalt-zinc-cadmium resistance protein CzcB, putative	0.0
T1E_5272	CzcC2	Me^2^	OEP	Cation efflux	Cobalt-zinc-cadmium resistance protein CzcC, putative	0.0
T1E_5277	ompR		Response regulator	Copper homeostasis, Cobalt-zinc-cadmium resistance	DNA-binding heavy metal response regulator	6e-124
T1E_5753	CopB1	Cu	OM protein	Cu chelation(?)	Copper resistance B precursor	e-159

Up to three different systems potentially involved in simultaneous cobalt, zinc and cadmium resistance were found. One of the cation efflux systems is the CzcD (T1E_2808) immersed in a cluster with the corresponding response regulator CzcR (T1E_2811) and the sensor histidine kinase encoded by the *czcS* gene (T1E_2812). Another family of transporters that may mediate the extrusion of these three heavy metal ions are the one encoded by the *cadA1* (T1E_2820) and *cadA2* (T1E_4489) genes; as well as by the resistance-nodulation-cell division (RND) pump CzcABC (T1E_5270, T1E_5271, T1E_5272). The CusABC efflux system (T1E_4694, T1E_4695, T1E_4696) is involved resistance to silver and copper ions. Seven genes involved in resistance to arsenite–arsenate–antimonite efflux were annotated. Four of them *arsHCBR* made an operon (T1E_2719–2722), and the three other genes related to arsenite resistance (T1E_4939, T1E_4996 and T1E_1144) are scattered throughout the genome. Finally one chromate resistance protein ChrA (T1E_3354) was found in the genome of T1E suggesting it is the responsible for chromate efflux in this strain.

## Biotransformation potential

As mentioned above DOT-T1E has the ability to thrive in the presence of toxic organic solvents that normally form a biphasic system with water. This property can be exploited to develop double-phase biotransformation systems (organic solvent and water) in which water insoluble chemicals, toxic substrates or chemical products are kept in the organic phase. The main advantages of these systems are that the product(s) is(are) continuously removed by a solvent phase, their toxic effects are decreased and the lifespan of the biocatalytic system is longer. In addition, if the concentration of the product increases in the organic phase, product recovery is easier and less costly (Bruce and Daugulis, 1991; Leon *et al*., 1998). Rojas and colleagues (2004) demonstrated that *P. putida* DOT-T1E was tolerant to different aliphatic alcohols such as decanol, nonanol and octanol. These aliphatic alcohols are useful in double-phase biotransformation systems to deliver hydrophobic or toxic compounds or to recover added value products that partition preferentially in the organic phase. This concept was exploited by Neumann and colleagues (2005) who showed that DOT-T1E in the presence of 1-decanol tolerated up to 200-fold higher concentrations of the model substrate 3-nitrotoluene than in aqueous medium. In the same line Wierckx and colleagues (2008) showed that phenol production from glucose by *P. putida* S12, another solvent tolerant strain, increased up to 10-fold using a biphasic system. This set of results is the basis that support the potential of DOT-T1E as a useful biocatalyst for biphasic systems.

As described above, a wide range of oxido-reductase enzymes are encoded in the genome of DOT-T1E, a number of which are of commercial interest. Among these are a numerous dioxygenases that might selectively hydroxylate the aromatic rings at positions 1 and 2; 2 and 3; 2 and 5; 3 and 4; and 4 and 5 (see Table S3). These dioxygenases may catalyse the stereo-specific dioxygenation of hydrocarbons and could yield secondary commodity chemicals such as adipic acid and γ-caprolactam.

At least 16 monooxygenases that may act on diverse chemicals have also been annotated (Table S4). Some of these enzymes have the potential to oxidize alkanes to their pertinent alcohols, and are of interest to generate added-value products such as linear branched alcohols, aromatic alcohols, diols, hydroxypropionic acid and others. Since the produced chemicals are not metabolized by *P. putida* DOT-T1E, they accumulate in culture supernatants and high yields can be achieved via extraction of the second phase (Fig. S8).

Biotransformations based on genetically engineering production strains for certain compounds may require either blocking an existing pathway, recruiting new enzymes, or a combination of both approaches. Some of these approaches have been used before with DOT-T1E or related solvent-tolerant strains. An example of biotransformation through inhibition of a single gene is the production of 3-methylcatechol from toluene. Thus, Rojas and colleagues (2004) showed that a catechol 2,3-dioxygenase knockout mutant of T1E in a 1-octanol/water bioreactor produced 20-fold higher amounts of the compound than in the aqueous medium; these results demonstrate the usefulness of double-phase systems.

Ramos-González and colleagues (2001) developed a system for transformation of toluene into 4-hydroxybenzoate which involves the use of a double mutant of T1E in which the toluene dioxygenase and *p*-hydroxybenzoate hydroxylase genes were first inactivated. Then, a set of genes for sequential oxidation of toluene via toluene 4-monooxygenase were incorporated and the recombinant strain system produced up to 35 mM of the product.

**Figure 5:**
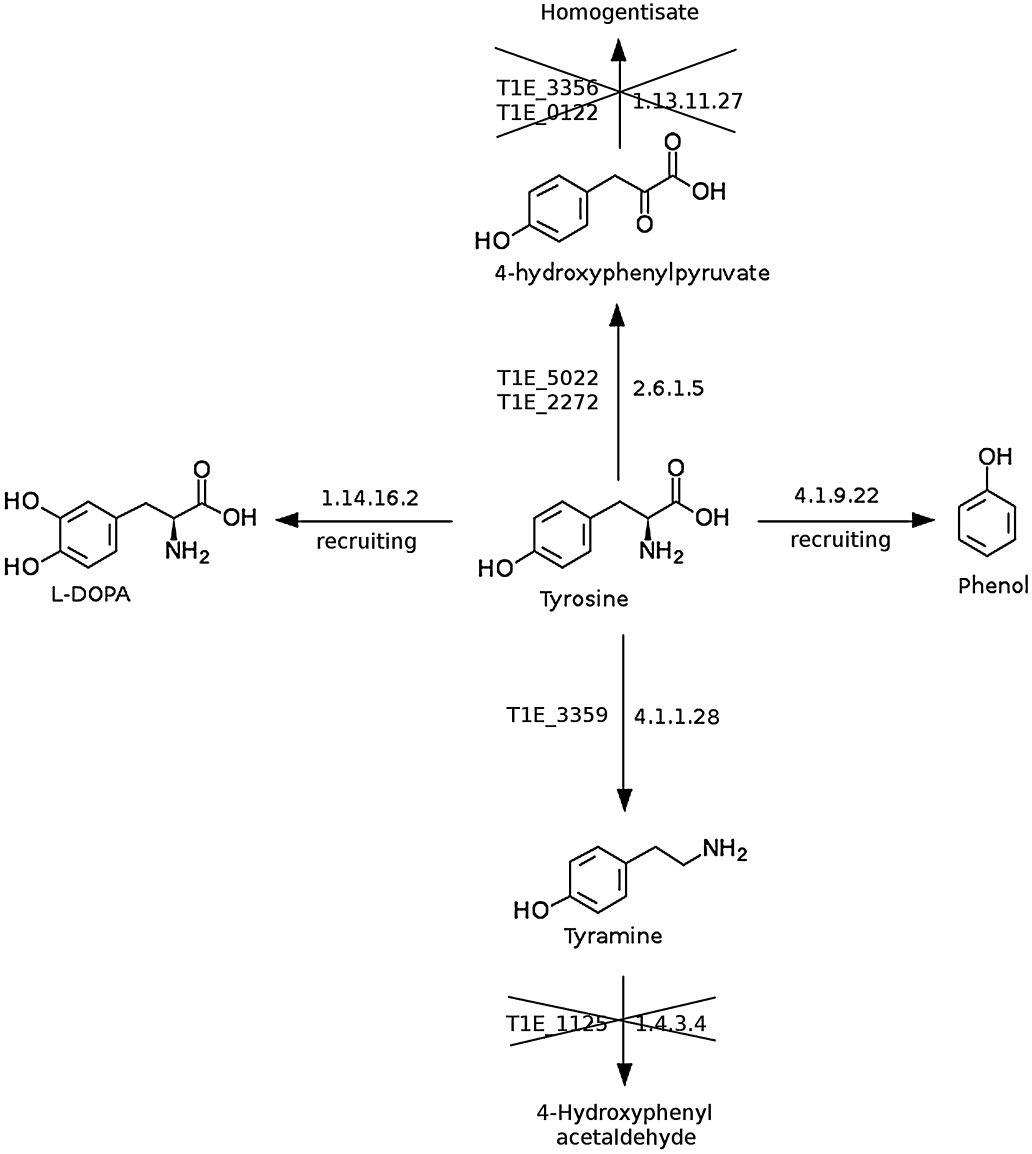
Biotransformation of tyrosine by *P. putida* through metabolic blockage or gene recruitment. The EC XXXX of the enzymes needed for the listed biotransformation are indicated. The text describes the approaches used by different research groups to achieve the indicated products.

Efficient bioconversion of glucose to phenol or cinnamic acid was achieved by Wierckx and colleagues (2005) and Nijkamp and colleagues (2005), respectively, with *P. putida* S12 to this end a tyrosine phenol lyase (EC 4.1.9.22) from *Pantoea agglomerans* was recruited and it enabled the S12 strain to produce phenol (Fig. 5). In fed-batch assays in water, the productivity was limited by accumulation of 5 mM phenol in the medium; above this concentration phenol was toxic. However, this toxicity was overcome by use of 1-octanol as a second phase and as an extractant for phenol in a biphasic system. This approach resulted in accumulation of nearly 50 mM phenol in the octanol phase (Wierckx *et al*., 2008). Other possibilities for the production of added-value molecules with DOT-T1E are their synthesis from tyrosine; for example, DOT-T1E can produce *L*-DOPA from tyrosine (Fig. 5). This can be achieved by recruiting one of the following activities: a polyphenol oxidase (EC 1.10.3.1), a tyrosinase (1.14.18.1) or a tyrosine 3-monooxygenase (E 1.14.16.2) (Krishnaveni *et al*., 2009; Surwase and Jadhav, 2011). It should also be noted that with tyrosine as a substrate DOT-T1E can produce tyramine (via an internal aromatic amino acid decarboxylase, EC 4.1.1.28) and 4-hydroxyphenylpyruvate using a tyrosine amino transferase (EC 2.6.1.5). However, accumulation of the products of these biotransformations requires the inhibition of further catabolism of the products because they can be used as a C source by DOT-T1E (Daniels *et al*., 2010).

**Figure 6:**
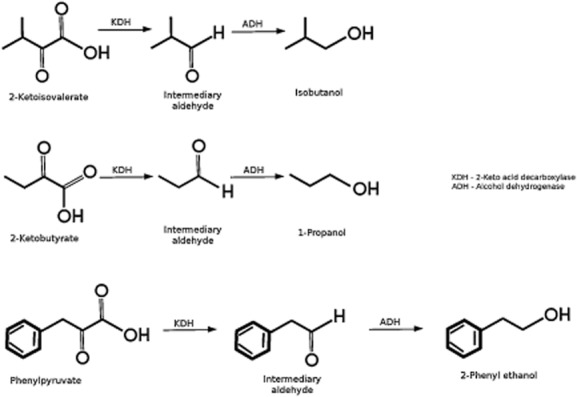
Potential synthesis of different alcohols from keto acids by DOT-T1E. 2-Ketoisovalerate, 2-ketobutyrate and phenylpyruvate are produced in the catabolism of isoleucine, threonine and tryptophane respectively. According to Atsumi and colleagues (2008) recruitment of a broad substrate range keto acid decarboxylase (KDH) yields an aldehyde, which along with one of the multiple alcohol dehydrogenase enzymes encoded in the genome of this strain can lead to the synthesis of the corresponding alcohol (see Table S1).

One of our aims is to customize strains for the production of aromatic alcohols for biofuel production. In this regard DOT-T1E can be used to produce alkyl and aromatic alcohols (Fig. 6) through blocking the catabolic pathways for amino acid degradation, in which keto acid intermediates are converted into their corresponding alcohols, as reported for *Escherichia coli* (Atsumi *et al*., 2008) – a process that requires the recruitment of a keto acid decarboxylase to produce an intermediate aldehyde that is subsequently transformed into its corresponding alcohol.

*D*-xylose is the second most abundant sugar in lignocellulosic materials and its utilization by industrial organisms to produce biofuels and added-value aromatic compounds is of interest (Octave and Thomas, 2005). As described above, strains of the *P. putida* species cannot use pentose sugars, but this was overcome via the engineered addition of *xylAB* genes, which allow the conversion of *D*-xylose in *D*-xylulose and xylulose-5-P, to allow metabolism of *D*-xylose via the pentose phosphate pathway (Meijnen *et al*., [Bibr b34],[Bibr b35]). However, growth was rather slow and fast growers were isolated after enrichment in fermentors. In a recent omics-based study, the authors have shown that high yield growth involved inactivation of glucose dehydrogenase and rearrangement of central carbon catabolism to allow for more efficient decarboxylation of 6-phosphogluconate for the catabolism of the sugar via the pentose phosphate pathway (Meijnen *et al*., 2012). Since *P. putida* S12 is as tolerant to solvents as DOT-T1E (Segura *et al*., 2003), we hypothesize that a similar strain of DOT-T1E could be engineered.

In summary, the analysis of the genome of the solvent-tolerant DOT-T1E strain explains the catabolic potential of this microorganism in accordance with previously published physiological studies. The use of the Pathway Tool platform together with the identification of enzymes using the international EC codes not only support the metabolic reactions, but also provide an opportunity to design biotransformation reactions to produce value-added products in high concentration. Due to the proven ability of DOT-T1E to thrive in the presence of a second organic phase in biphasic systems, only minimal genetic manipulation will be required in order to reap substantial reward from the genomic analysis reported here.

## Supporting information

**Text S1.** Details on enzyme screens. **Fig. S1.** Catabolism of glucose as an example of sugar metabolism based on annotated genes. Glucose can be oxidized in the periplasm to yield gluconate or ketogluconate or in the cytoplasm upon phosphorylation. All three pathways converge at the level of 6-phosphogluconate that is metabolized via the Entner–Doudoroff pathway that yields chemicals that feed the Krebs cycle. The genes are annotated according to del Castillo *et al*. (2007). **Fig. S2.** Catabolism of *L*- and *D*-lysine as an example of an amino acid being used as a source of carbon through multiple confluent pathways. The set of genes and most of the intermediates were analysed in detail by Revelles and colleagues (2007) for the KT2440 strain. All genes shown in this pathway for DOT-T1E are based on the identification of the homologous genes. **Fig. S3.** Catabolism of lactic acid by P. *putida* DOT-T1E as an example of the metabolism of an organic acid. Information was derived from Nelson and colleagues (2002). Lactate enables the growth of T1E in minimal medium with doubling times in the range of 120 ± 10 min. Inactivation of the *lldD* gene blocks the use of lactate as a C source. **Fig. S4.** Catabolism of aromatic compounds to a set of central intermediates. For the described reaction the corresponding enzyme(s) were identified as homologous to those described in detail by Jiménez and colleagues (2002) for KT2440 strains. A set of peripheral enzymes lead to the formation of a number of catechol-related compounds, which upon ortho or meta-cleavage yielded Krebs cycle intermediates. **Fig. S5.** Genetic organization of the genes for the protocatechuete central catabolic pathway. The genes were identified by BLAST analysis and the operon structure of the genes deduced from the overlapping nature of all the genes, except pcaF and pcaT; however, these genes form an operon based on RT-PCR analysis with RNA isolated from strains grown in 4-hydroxybenzoate (unpublished). **Fig. S6.** Genetic organization of the duplicated cat genes located in two different chromosomal regions. One of the cat clusters includes the catR gene that probably controls the expression of the two *catABC* operons based on high sequence conservation of the promoter region in the two catB genes. **Fig. S7.** Genes and enzymes for the utilization of methionine as an S source by DOT-T1E. The gene products involved in the catabolism of methionine were identified based on BLAST analyses. **Fig. S8.** Set of biotransformation reactions to achieve the synthesis of adipate/3-oxoadipyl-CoA based on gene content in DOT-T1E strain. Multiple steps are represented by X^1^, X^2^ and X^3^. The loci are: X^1^. – T1E-0314, T1E-0333, T1E-0690, T1E-0898, T1E-1004, T1E-1466, T1E1534, T1E-2226, T1E-2233, T1E-2397, T1E-3843, T1E-3853, T1E-3860, T1E-4416, T1E-5253, T1E-5478; X^2^. – T1E-0103, T1E-1864, T1E-1899, T1E-2485, T1E-2953, T1E-3631, T1E-4716, T1E-4861, T1E-4862; X^3^. – T1E-0369, T1E-1262, T1E-4182, T1E-4445. **Table S1.** *Pseudomonas putida* DOT-T1E enzymes of according to four digits EC classification. **Table S2.** C, N and S sources identified in *silico* and used by *P. putida* DOT-T1E confirmed experimentally. **Table S3.** Dioxygenases encoded by the *P. putida* DOT-T1E genome. **Table S4.** Monooxygenases encoded by the *P. putida* DOTT1E genome.
