# Supplementary figures and images for: Metabolic potential of the organic-solvent tolerant Pseudomonas putida DOT-T1E deduced from its annotated genome
Source: Microb Biotechnol. 2013 Jul 1;6(5):598–611. doi: 10.1111/1751-7915.12061 (PMC3918161; doi:10.1111/1751-7915.12061)

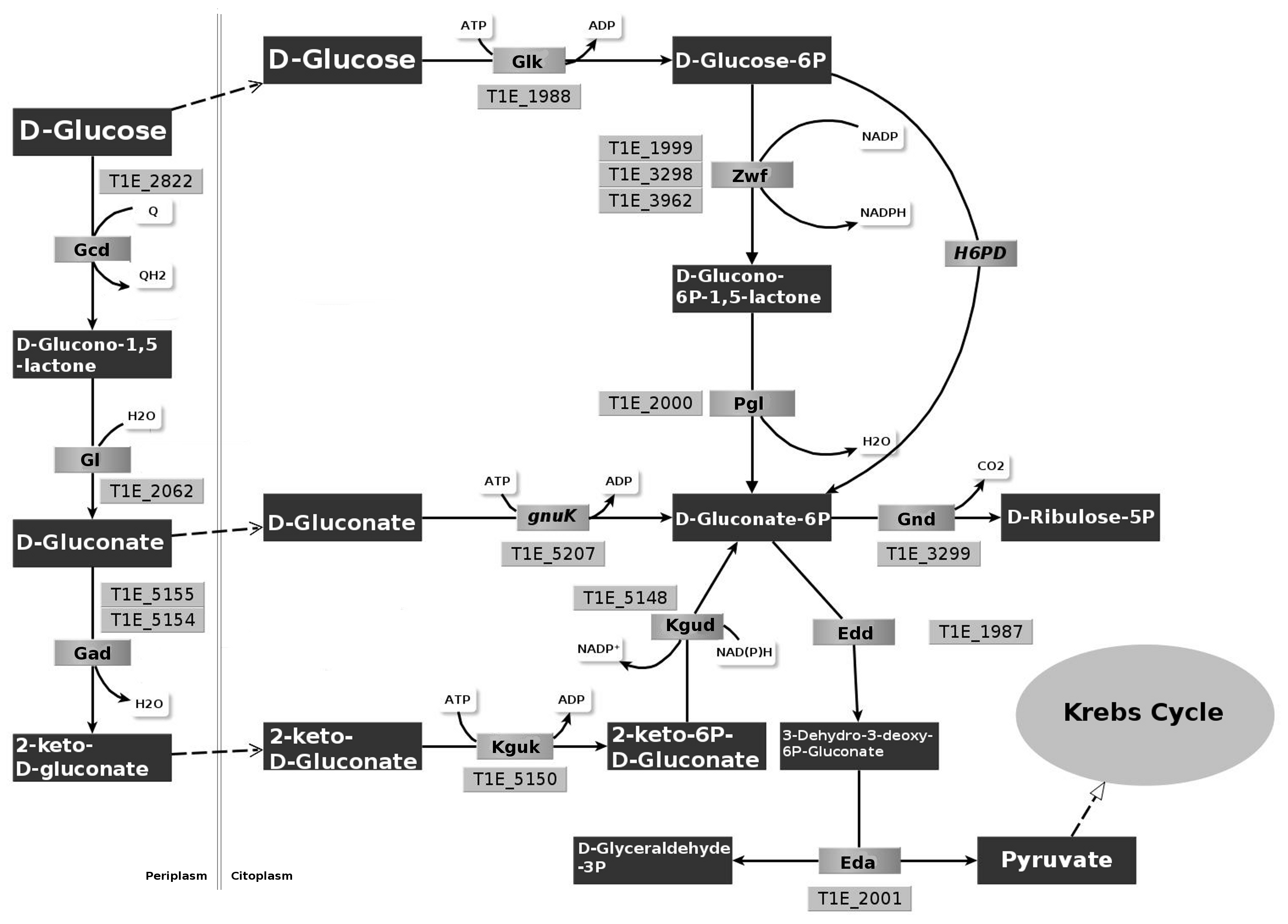

Supplement: Supplementary file 1 — Text S1. Details on enzyme screens. Fig. S1. Catabolism of glucose as an example of sugar metabolism based on annotated genes. Glucose can be oxidized in the periplasm to yield gluconate or ketogluconate or in the cytoplasm upon phosphorylation. All three pathways converge at the level of 6-phosphogluconate that is metabolized via the Entner–Doudoroff pathway that yields chemicals that feed the Krebs cycle. The genes are annotated according to del Castillo et al. (2007). Fig. S2. Catabolism of L- and D-lysine as an example of an amino acid being used as a source of carbon through multiple confluent pathways. The set of genes and most of the intermediates were analysed in detail by Revelles and colleagues (2007) for the KT2440 strain. All genes shown in this pathway for DOT-T1E are based on the identification of the homologous genes. Fig. S3. Catabolism of lactic acid by P. putida DOT-T1E as an example of the metabolism of an organic acid. Information was derived from Nelson and colleagues (2002). Lactate enables the growth of T1E in minimal medium with doubling times in the range of 120 ± 10 min. Inactivation of the lldD gene blocks the use of lactate as a C source. Fig. S4. Catabolism of aromatic compounds to a set of central intermediates. For the described reaction the corresponding enzyme(s) were identified as homologous to those described in detail by Jiménez and colleagues (2002) for KT2440 strains. A set of peripheral enzymes lead to the formation of a number of catechol-related compounds, which upon ortho or meta-cleavage yielded Krebs cycle intermediates. Fig. S5. Genetic organization of the genes for the protocatechuete central catabolic pathway. The genes were identified by BLAST analysis and the operon structure of the genes deduced from the overlapping nature of all the genes, except pcaF and pcaT; however, these genes form an operon based on RT-PCR analysis with RNA isolated from strains grown in 4-hydroxybenzoate (unpublished). Fig. S6. Genetic organi [file mbt0006-0598-sd1.zip › mbt2_12061_sm_figureS1.tif]

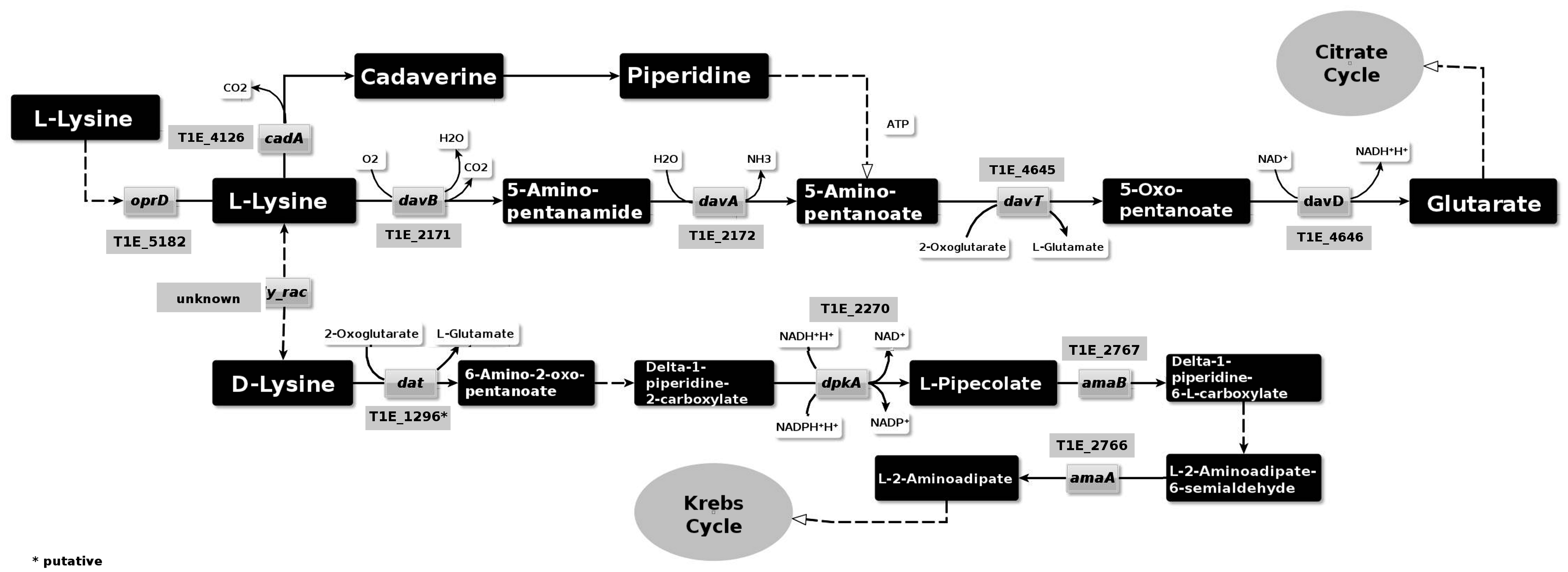

Supplement: Supplementary file 1 — Text S1. Details on enzyme screens. Fig. S1. Catabolism of glucose as an example of sugar metabolism based on annotated genes. Glucose can be oxidized in the periplasm to yield gluconate or ketogluconate or in the cytoplasm upon phosphorylation. All three pathways converge at the level of 6-phosphogluconate that is metabolized via the Entner–Doudoroff pathway that yields chemicals that feed the Krebs cycle. The genes are annotated according to del Castillo et al. (2007). Fig. S2. Catabolism of L- and D-lysine as an example of an amino acid being used as a source of carbon through multiple confluent pathways. The set of genes and most of the intermediates were analysed in detail by Revelles and colleagues (2007) for the KT2440 strain. All genes shown in this pathway for DOT-T1E are based on the identification of the homologous genes. Fig. S3. Catabolism of lactic acid by P. putida DOT-T1E as an example of the metabolism of an organic acid. Information was derived from Nelson and colleagues (2002). Lactate enables the growth of T1E in minimal medium with doubling times in the range of 120 ± 10 min. Inactivation of the lldD gene blocks the use of lactate as a C source. Fig. S4. Catabolism of aromatic compounds to a set of central intermediates. For the described reaction the corresponding enzyme(s) were identified as homologous to those described in detail by Jiménez and colleagues (2002) for KT2440 strains. A set of peripheral enzymes lead to the formation of a number of catechol-related compounds, which upon ortho or meta-cleavage yielded Krebs cycle intermediates. Fig. S5. Genetic organization of the genes for the protocatechuete central catabolic pathway. The genes were identified by BLAST analysis and the operon structure of the genes deduced from the overlapping nature of all the genes, except pcaF and pcaT; however, these genes form an operon based on RT-PCR analysis with RNA isolated from strains grown in 4-hydroxybenzoate (unpublished). Fig. S6. Genetic organi [file mbt0006-0598-sd1.zip › mbt2_12061_sm_figureS2.tif]

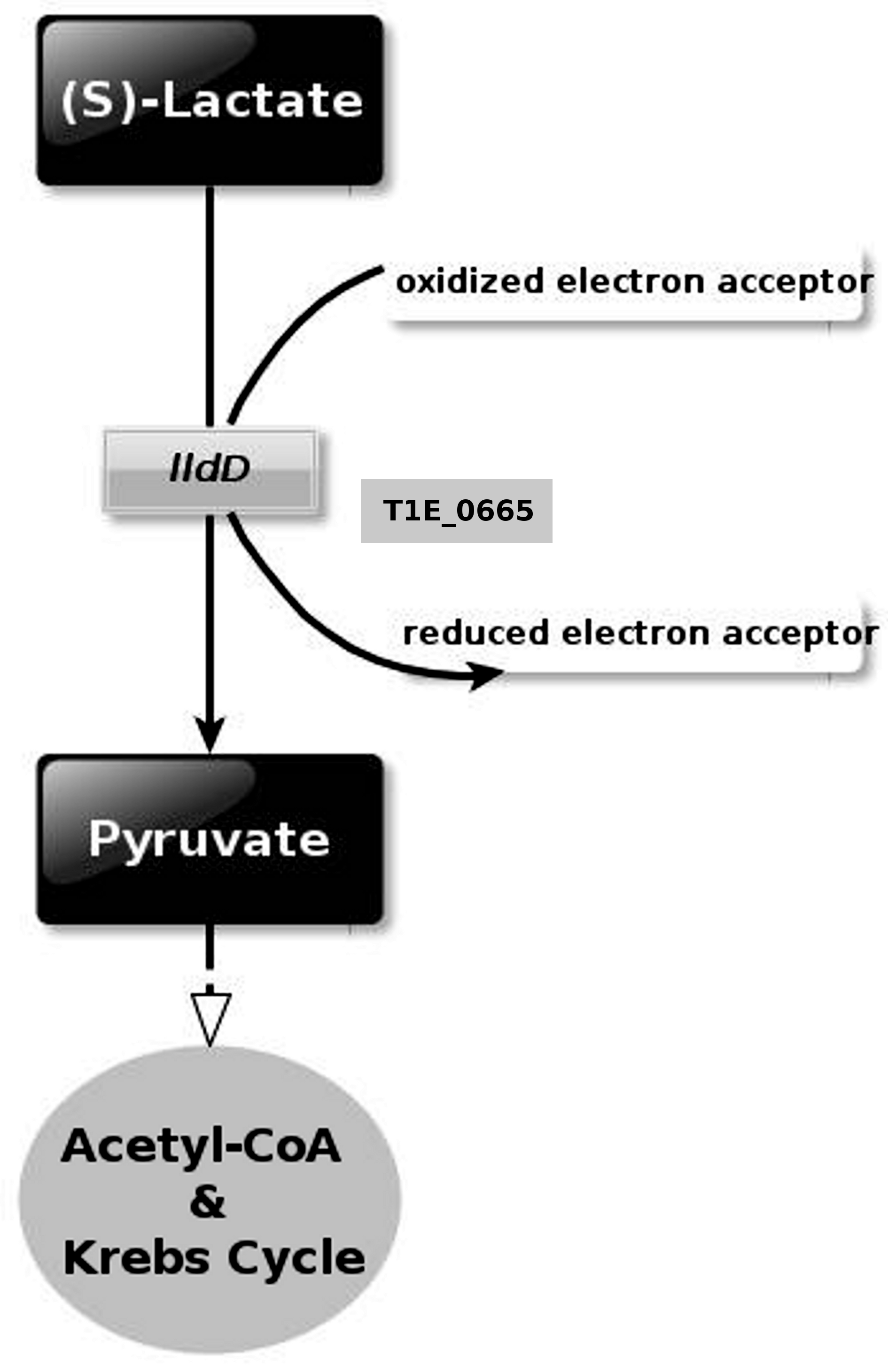

Supplement: Supplementary file 1 — Text S1. Details on enzyme screens. Fig. S1. Catabolism of glucose as an example of sugar metabolism based on annotated genes. Glucose can be oxidized in the periplasm to yield gluconate or ketogluconate or in the cytoplasm upon phosphorylation. All three pathways converge at the level of 6-phosphogluconate that is metabolized via the Entner–Doudoroff pathway that yields chemicals that feed the Krebs cycle. The genes are annotated according to del Castillo et al. (2007). Fig. S2. Catabolism of L- and D-lysine as an example of an amino acid being used as a source of carbon through multiple confluent pathways. The set of genes and most of the intermediates were analysed in detail by Revelles and colleagues (2007) for the KT2440 strain. All genes shown in this pathway for DOT-T1E are based on the identification of the homologous genes. Fig. S3. Catabolism of lactic acid by P. putida DOT-T1E as an example of the metabolism of an organic acid. Information was derived from Nelson and colleagues (2002). Lactate enables the growth of T1E in minimal medium with doubling times in the range of 120 ± 10 min. Inactivation of the lldD gene blocks the use of lactate as a C source. Fig. S4. Catabolism of aromatic compounds to a set of central intermediates. For the described reaction the corresponding enzyme(s) were identified as homologous to those described in detail by Jiménez and colleagues (2002) for KT2440 strains. A set of peripheral enzymes lead to the formation of a number of catechol-related compounds, which upon ortho or meta-cleavage yielded Krebs cycle intermediates. Fig. S5. Genetic organization of the genes for the protocatechuete central catabolic pathway. The genes were identified by BLAST analysis and the operon structure of the genes deduced from the overlapping nature of all the genes, except pcaF and pcaT; however, these genes form an operon based on RT-PCR analysis with RNA isolated from strains grown in 4-hydroxybenzoate (unpublished). Fig. S6. Genetic organi [file mbt0006-0598-sd1.zip › mbt2_12061_sm_figureS3.tif]

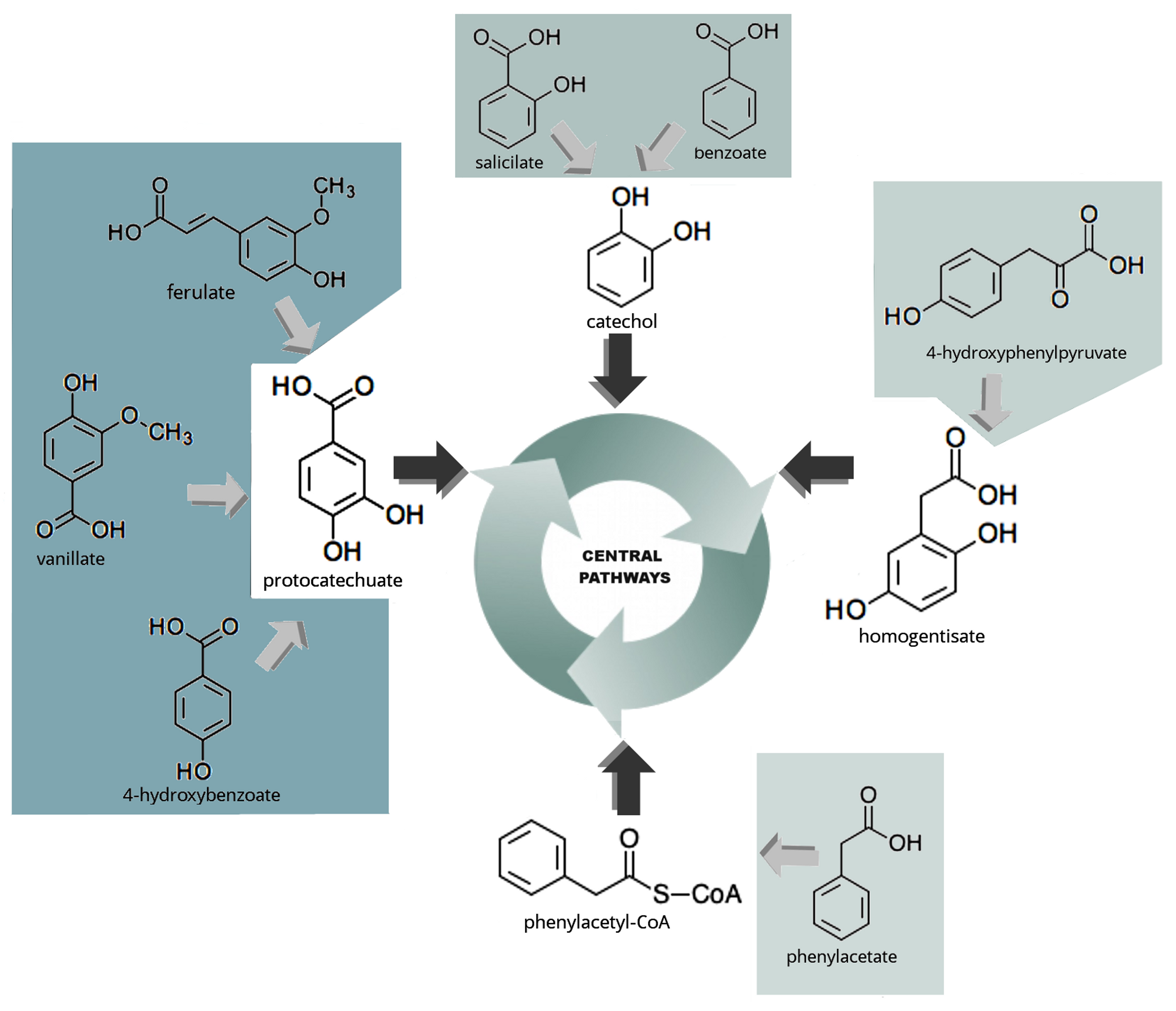

Supplement: Supplementary file 1 — Text S1. Details on enzyme screens. Fig. S1. Catabolism of glucose as an example of sugar metabolism based on annotated genes. Glucose can be oxidized in the periplasm to yield gluconate or ketogluconate or in the cytoplasm upon phosphorylation. All three pathways converge at the level of 6-phosphogluconate that is metabolized via the Entner–Doudoroff pathway that yields chemicals that feed the Krebs cycle. The genes are annotated according to del Castillo et al. (2007). Fig. S2. Catabolism of L- and D-lysine as an example of an amino acid being used as a source of carbon through multiple confluent pathways. The set of genes and most of the intermediates were analysed in detail by Revelles and colleagues (2007) for the KT2440 strain. All genes shown in this pathway for DOT-T1E are based on the identification of the homologous genes. Fig. S3. Catabolism of lactic acid by P. putida DOT-T1E as an example of the metabolism of an organic acid. Information was derived from Nelson and colleagues (2002). Lactate enables the growth of T1E in minimal medium with doubling times in the range of 120 ± 10 min. Inactivation of the lldD gene blocks the use of lactate as a C source. Fig. S4. Catabolism of aromatic compounds to a set of central intermediates. For the described reaction the corresponding enzyme(s) were identified as homologous to those described in detail by Jiménez and colleagues (2002) for KT2440 strains. A set of peripheral enzymes lead to the formation of a number of catechol-related compounds, which upon ortho or meta-cleavage yielded Krebs cycle intermediates. Fig. S5. Genetic organization of the genes for the protocatechuete central catabolic pathway. The genes were identified by BLAST analysis and the operon structure of the genes deduced from the overlapping nature of all the genes, except pcaF and pcaT; however, these genes form an operon based on RT-PCR analysis with RNA isolated from strains grown in 4-hydroxybenzoate (unpublished). Fig. S6. Genetic organi [file mbt0006-0598-sd1.zip › mbt2_12061_sm_figureS4.tif]

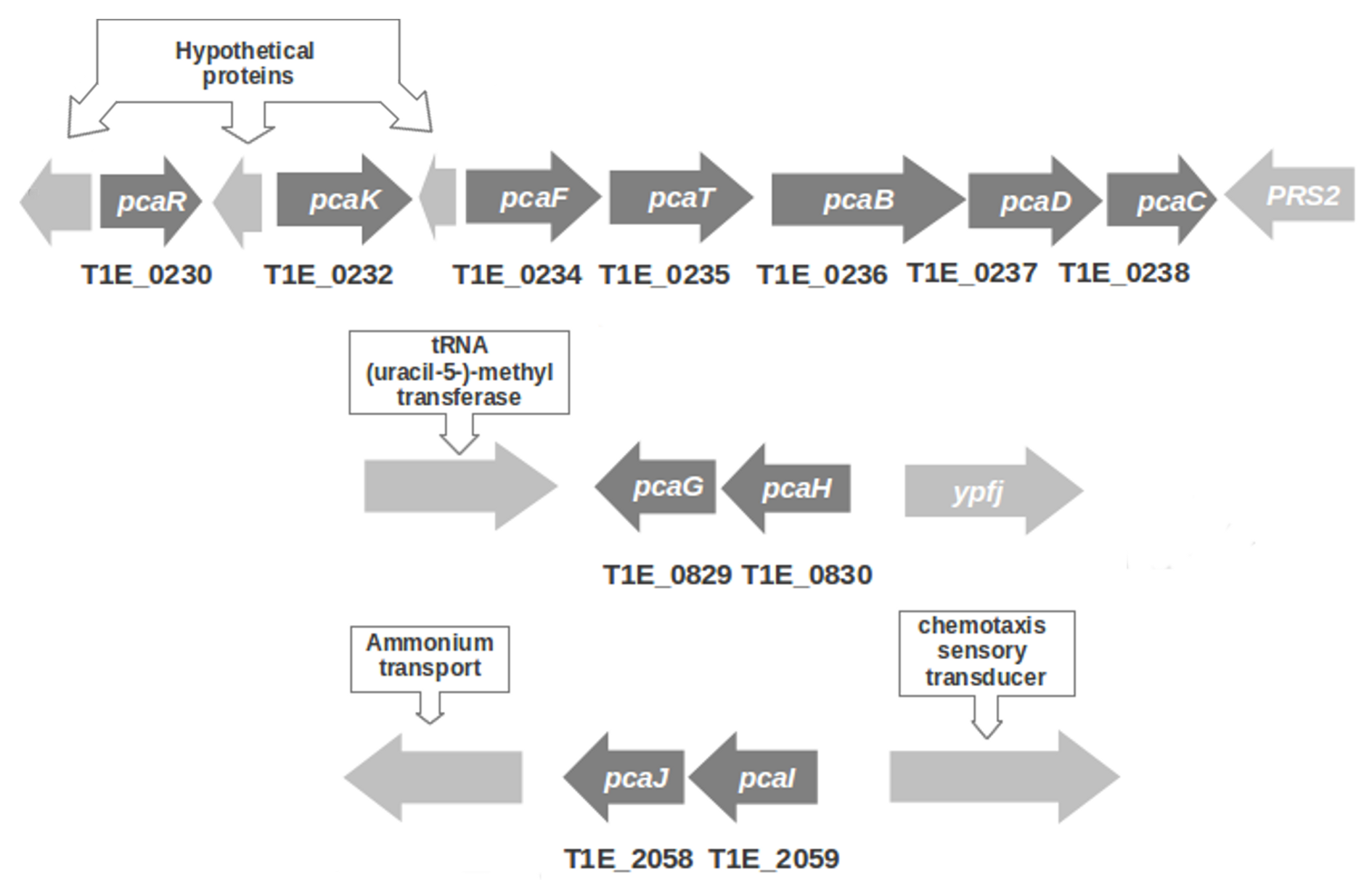

Supplement: Supplementary file 1 — Text S1. Details on enzyme screens. Fig. S1. Catabolism of glucose as an example of sugar metabolism based on annotated genes. Glucose can be oxidized in the periplasm to yield gluconate or ketogluconate or in the cytoplasm upon phosphorylation. All three pathways converge at the level of 6-phosphogluconate that is metabolized via the Entner–Doudoroff pathway that yields chemicals that feed the Krebs cycle. The genes are annotated according to del Castillo et al. (2007). Fig. S2. Catabolism of L- and D-lysine as an example of an amino acid being used as a source of carbon through multiple confluent pathways. The set of genes and most of the intermediates were analysed in detail by Revelles and colleagues (2007) for the KT2440 strain. All genes shown in this pathway for DOT-T1E are based on the identification of the homologous genes. Fig. S3. Catabolism of lactic acid by P. putida DOT-T1E as an example of the metabolism of an organic acid. Information was derived from Nelson and colleagues (2002). Lactate enables the growth of T1E in minimal medium with doubling times in the range of 120 ± 10 min. Inactivation of the lldD gene blocks the use of lactate as a C source. Fig. S4. Catabolism of aromatic compounds to a set of central intermediates. For the described reaction the corresponding enzyme(s) were identified as homologous to those described in detail by Jiménez and colleagues (2002) for KT2440 strains. A set of peripheral enzymes lead to the formation of a number of catechol-related compounds, which upon ortho or meta-cleavage yielded Krebs cycle intermediates. Fig. S5. Genetic organization of the genes for the protocatechuete central catabolic pathway. The genes were identified by BLAST analysis and the operon structure of the genes deduced from the overlapping nature of all the genes, except pcaF and pcaT; however, these genes form an operon based on RT-PCR analysis with RNA isolated from strains grown in 4-hydroxybenzoate (unpublished). Fig. S6. Genetic organi [file mbt0006-0598-sd1.zip › mbt2_12061_sm_figureS5.tif]

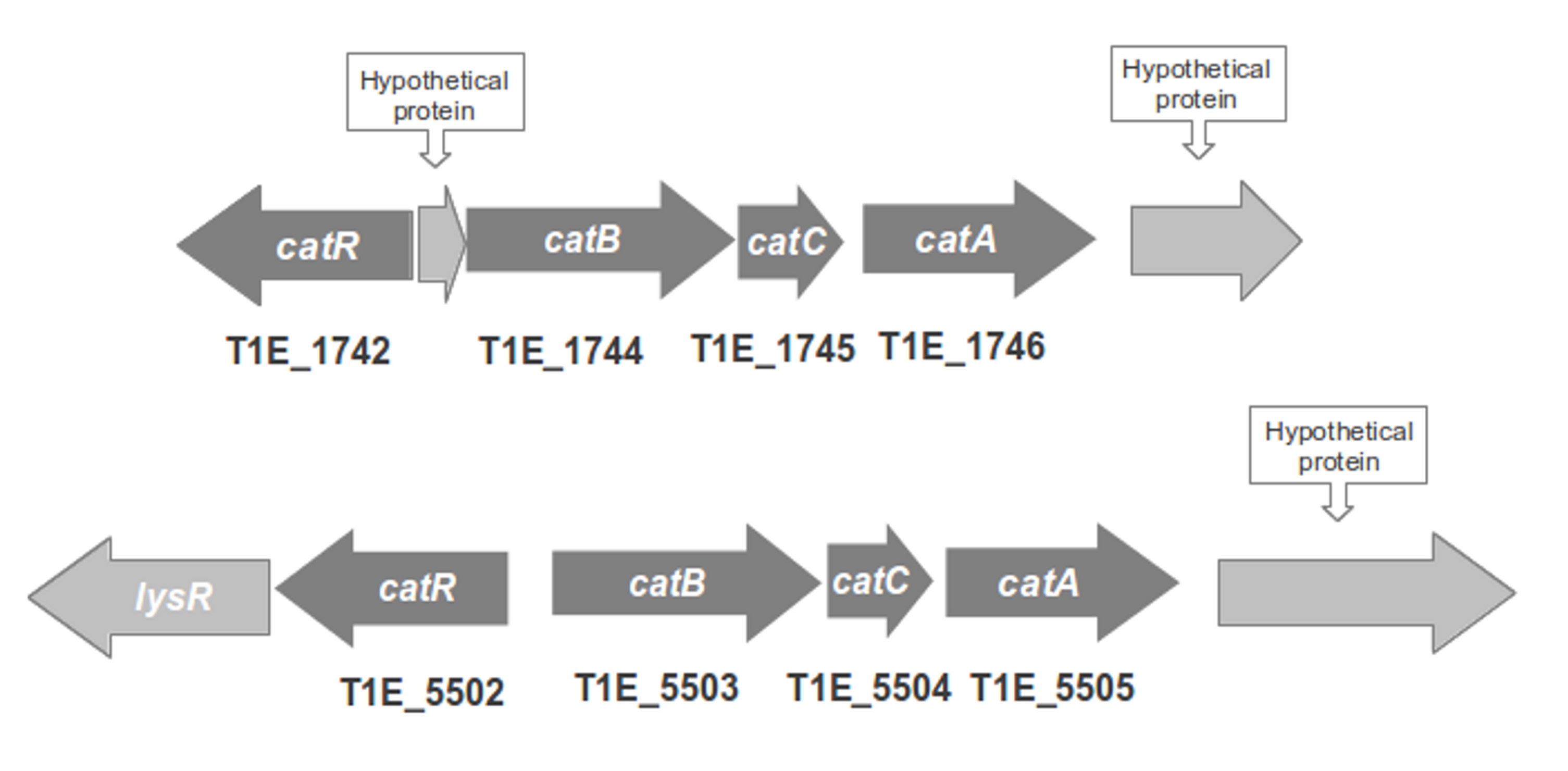

Supplement: Supplementary file 1 — Text S1. Details on enzyme screens. Fig. S1. Catabolism of glucose as an example of sugar metabolism based on annotated genes. Glucose can be oxidized in the periplasm to yield gluconate or ketogluconate or in the cytoplasm upon phosphorylation. All three pathways converge at the level of 6-phosphogluconate that is metabolized via the Entner–Doudoroff pathway that yields chemicals that feed the Krebs cycle. The genes are annotated according to del Castillo et al. (2007). Fig. S2. Catabolism of L- and D-lysine as an example of an amino acid being used as a source of carbon through multiple confluent pathways. The set of genes and most of the intermediates were analysed in detail by Revelles and colleagues (2007) for the KT2440 strain. All genes shown in this pathway for DOT-T1E are based on the identification of the homologous genes. Fig. S3. Catabolism of lactic acid by P. putida DOT-T1E as an example of the metabolism of an organic acid. Information was derived from Nelson and colleagues (2002). Lactate enables the growth of T1E in minimal medium with doubling times in the range of 120 ± 10 min. Inactivation of the lldD gene blocks the use of lactate as a C source. Fig. S4. Catabolism of aromatic compounds to a set of central intermediates. For the described reaction the corresponding enzyme(s) were identified as homologous to those described in detail by Jiménez and colleagues (2002) for KT2440 strains. A set of peripheral enzymes lead to the formation of a number of catechol-related compounds, which upon ortho or meta-cleavage yielded Krebs cycle intermediates. Fig. S5. Genetic organization of the genes for the protocatechuete central catabolic pathway. The genes were identified by BLAST analysis and the operon structure of the genes deduced from the overlapping nature of all the genes, except pcaF and pcaT; however, these genes form an operon based on RT-PCR analysis with RNA isolated from strains grown in 4-hydroxybenzoate (unpublished). Fig. S6. Genetic organi [file mbt0006-0598-sd1.zip › mbt2_12061_sm_figureS6.tiff]

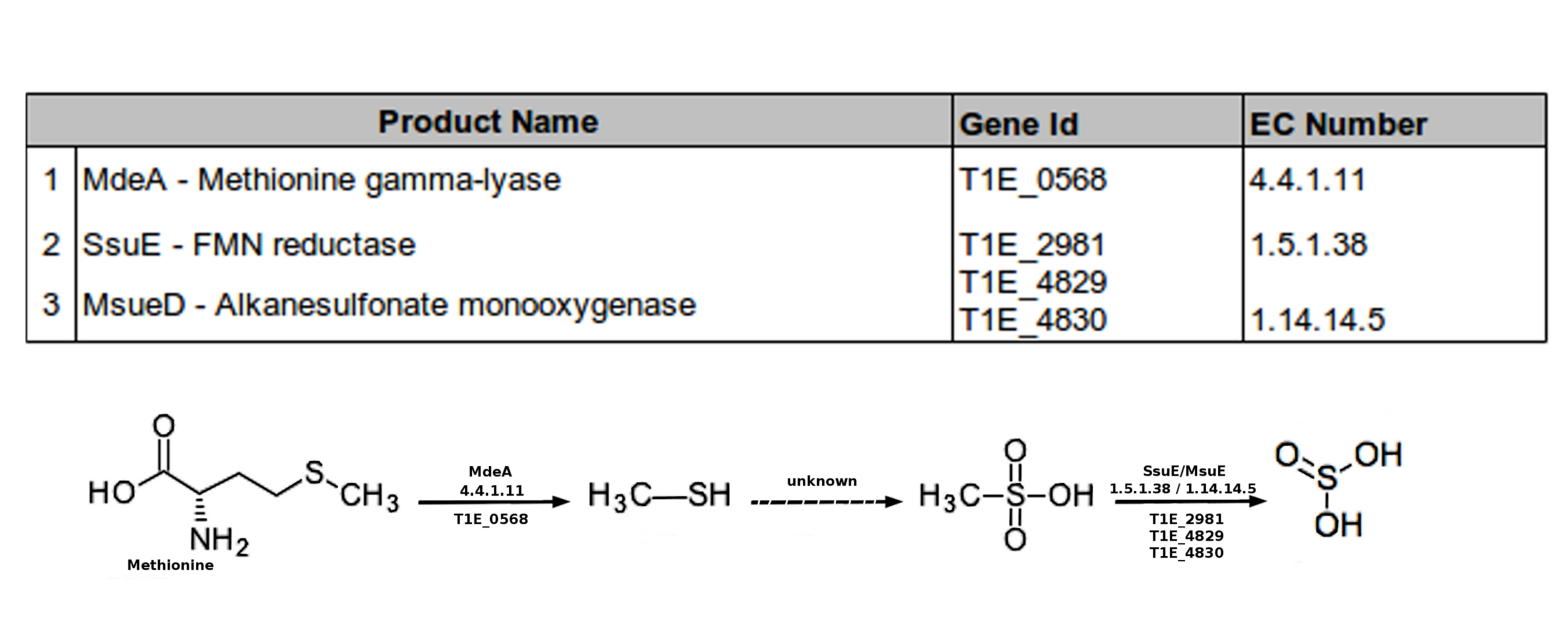

Supplement: Supplementary file 1 — Text S1. Details on enzyme screens. Fig. S1. Catabolism of glucose as an example of sugar metabolism based on annotated genes. Glucose can be oxidized in the periplasm to yield gluconate or ketogluconate or in the cytoplasm upon phosphorylation. All three pathways converge at the level of 6-phosphogluconate that is metabolized via the Entner–Doudoroff pathway that yields chemicals that feed the Krebs cycle. The genes are annotated according to del Castillo et al. (2007). Fig. S2. Catabolism of L- and D-lysine as an example of an amino acid being used as a source of carbon through multiple confluent pathways. The set of genes and most of the intermediates were analysed in detail by Revelles and colleagues (2007) for the KT2440 strain. All genes shown in this pathway for DOT-T1E are based on the identification of the homologous genes. Fig. S3. Catabolism of lactic acid by P. putida DOT-T1E as an example of the metabolism of an organic acid. Information was derived from Nelson and colleagues (2002). Lactate enables the growth of T1E in minimal medium with doubling times in the range of 120 ± 10 min. Inactivation of the lldD gene blocks the use of lactate as a C source. Fig. S4. Catabolism of aromatic compounds to a set of central intermediates. For the described reaction the corresponding enzyme(s) were identified as homologous to those described in detail by Jiménez and colleagues (2002) for KT2440 strains. A set of peripheral enzymes lead to the formation of a number of catechol-related compounds, which upon ortho or meta-cleavage yielded Krebs cycle intermediates. Fig. S5. Genetic organization of the genes for the protocatechuete central catabolic pathway. The genes were identified by BLAST analysis and the operon structure of the genes deduced from the overlapping nature of all the genes, except pcaF and pcaT; however, these genes form an operon based on RT-PCR analysis with RNA isolated from strains grown in 4-hydroxybenzoate (unpublished). Fig. S6. Genetic organi [file mbt0006-0598-sd1.zip › mbt2_12061_sm_figureS7.tiff]

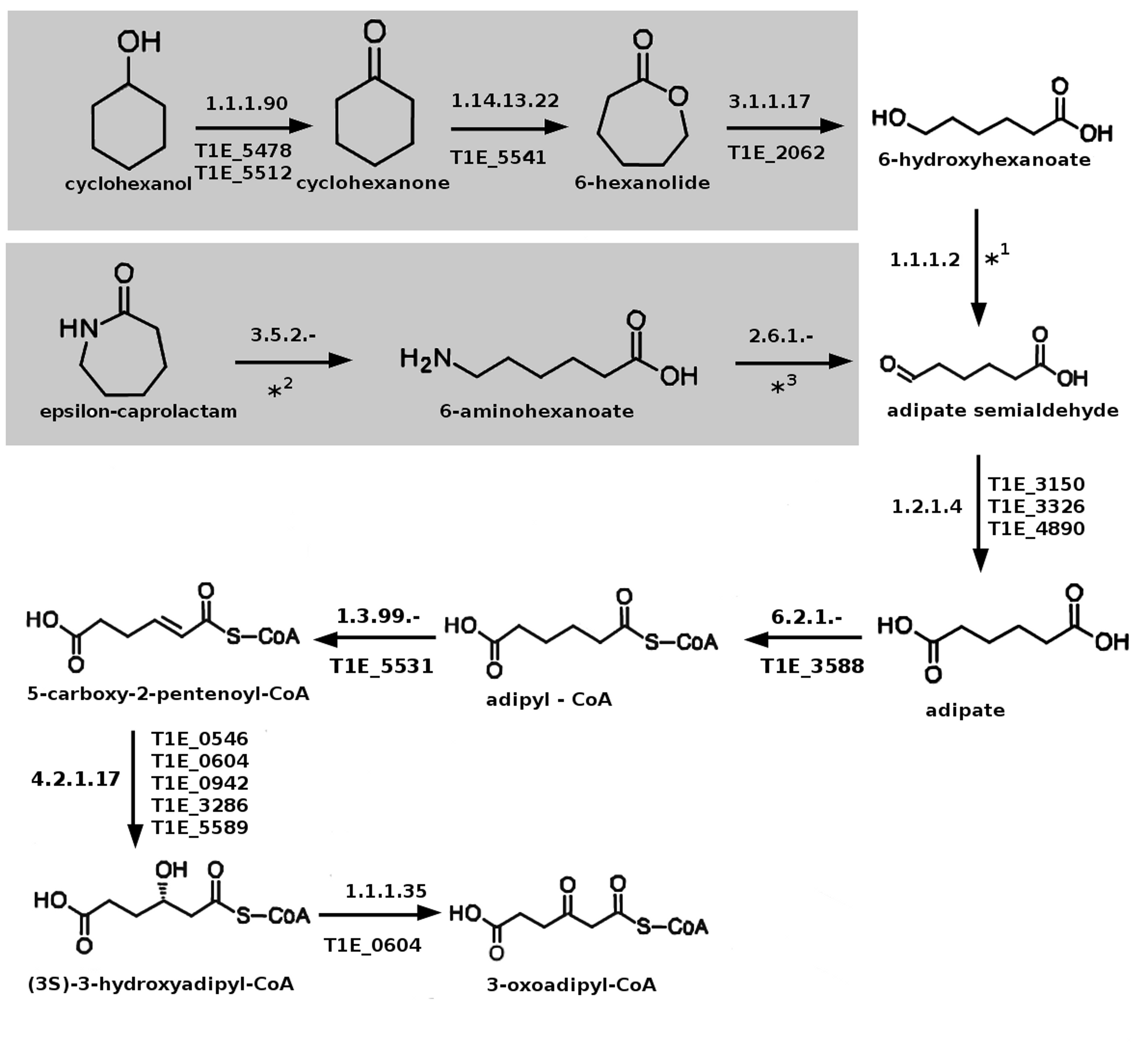

Supplement: Supplementary file 1 — Text S1. Details on enzyme screens. Fig. S1. Catabolism of glucose as an example of sugar metabolism based on annotated genes. Glucose can be oxidized in the periplasm to yield gluconate or ketogluconate or in the cytoplasm upon phosphorylation. All three pathways converge at the level of 6-phosphogluconate that is metabolized via the Entner–Doudoroff pathway that yields chemicals that feed the Krebs cycle. The genes are annotated according to del Castillo et al. (2007). Fig. S2. Catabolism of L- and D-lysine as an example of an amino acid being used as a source of carbon through multiple confluent pathways. The set of genes and most of the intermediates were analysed in detail by Revelles and colleagues (2007) for the KT2440 strain. All genes shown in this pathway for DOT-T1E are based on the identification of the homologous genes. Fig. S3. Catabolism of lactic acid by P. putida DOT-T1E as an example of the metabolism of an organic acid. Information was derived from Nelson and colleagues (2002). Lactate enables the growth of T1E in minimal medium with doubling times in the range of 120 ± 10 min. Inactivation of the lldD gene blocks the use of lactate as a C source. Fig. S4. Catabolism of aromatic compounds to a set of central intermediates. For the described reaction the corresponding enzyme(s) were identified as homologous to those described in detail by Jiménez and colleagues (2002) for KT2440 strains. A set of peripheral enzymes lead to the formation of a number of catechol-related compounds, which upon ortho or meta-cleavage yielded Krebs cycle intermediates. Fig. S5. Genetic organization of the genes for the protocatechuete central catabolic pathway. The genes were identified by BLAST analysis and the operon structure of the genes deduced from the overlapping nature of all the genes, except pcaF and pcaT; however, these genes form an operon based on RT-PCR analysis with RNA isolated from strains grown in 4-hydroxybenzoate (unpublished). Fig. S6. Genetic organi [file mbt0006-0598-sd1.zip › mbt2_12061_sm_figureS8.tif]
