# Supplementary material for: Metabolic potential of the organic-solvent tolerant Pseudomonas putida DOT-T1E deduced from its annotated genome
Source: Microb Biotechnol. 2013 Jul 1;6(5):598–611. doi: 10.1111/1751-7915.12061 (PMC3918161; doi:10.1111/1751-7915.12061)
Supplement: Supplementary file 1 — Text S1. Details on enzyme screens. Fig. S1. Catabolism of glucose as an example of sugar metabolism based on annotated genes. Glucose can be oxidized in the periplasm to yield gluconate or ketogluconate or in the cytoplasm upon phosphorylation. All three pathways converge at the level of 6-phosphogluconate that is metabolized via the Entner–Doudoroff pathway that yields chemicals that feed the Krebs cycle. The genes are annotated according to del Castillo et al. (2007). Fig. S2. Catabolism of L- and D-lysine as an example of an amino acid being used as a source of carbon through multiple confluent pathways. The set of genes and most of the intermediates were analysed in detail by Revelles and colleagues (2007) for the KT2440 strain. All genes shown in this pathway for DOT-T1E are based on the identification of the homologous genes. Fig. S3. Catabolism of lactic acid by P. putida DOT-T1E as an example of the metabolism of an organic acid. Information was derived from Nelson and colleagues (2002). Lactate enables the growth of T1E in minimal medium with doubling times in the range of 120 ± 10 min. Inactivation of the lldD gene blocks the use of lactate as a C source. Fig. S4. Catabolism of aromatic compounds to a set of central intermediates. For the described reaction the corresponding enzyme(s) were identified as homologous to those described in detail by Jiménez and colleagues (2002) for KT2440 strains. A set of peripheral enzymes lead to the formation of a number of catechol-related compounds, which upon ortho or meta-cleavage yielded Krebs cycle intermediates. Fig. S5. Genetic organization of the genes for the protocatechuete central catabolic pathway. The genes were identified by BLAST analysis and the operon structure of the genes deduced from the overlapping nature of all the genes, except pcaF and pcaT; however, these genes form an operon based on RT-PCR analysis with RNA isolated from strains grown in 4-hydroxybenzoate (unpublished). Fig. S6. Genetic organi [file mbt0006-0598-sd1.zip › mbt2_12061_sm.doc]

**Legends for Supplementary Figures**

**Suppl. Figure 1**. Catabolism of glucose as an example of sugar metabolism based on annotated genes. Glucose can be oxidized in the periplasm to yield gluconate or ketogluconate or in the cytoplasm upon phosphorylation. All three pathways converge at the level of 6-phosphogluconate that is metabolized via the Entner-Doudoroff pathway that yields chemicals that feed the Krebs cycle. The genes are annotated according to del Castillo *et al*. (2007).

**Suppl. Figure 2**. Catabolism of *L*- and *D*-lysine as an example of an amino acid being used as a source of carbon through multiple confluent pathways. The set of genes and most of the intermediates were analyzed in detail by Revelles *et al*. (2007) for the KT2440 strain. All genes shown in this pathway for DOT-T1E are based on the identification of the homologous genes.

**Suppl. Figure 3**. Catabolism of lactic acid by *P. putida* DOT-T1E as an example of the metabolism of an organic acid. Information was derived from Nelson *et al*. (2002). Lactate enables the growth of T1E in minimal medium with doubling times in the range of 120±10 min.Inactivation of the *lldD* gene blocks the use of lactate as a C-source.

**Suppl. Figure 4**. Catabolism of aromatic compounds to a set of central intermediates. For the described reaction the corresponding enzyme(s) were identified as homologous to those described in detail by Jiménez *et al*. (2002) for KT2440 strains. A set of peripheral enzymes lead to the formation of a number of catechol-related compounds, which upon *ortho* or *meta*-cleavage yielded Krebs cycle intermediates.

**Suppl. Figure 5**. Genetic organization of the genes for the protocatechuete central catabolic pathway. The genes were identified by BLAST analysis and the operon structure of the genes deduced from the overlapping nature of all the genes, except *pcaF* and *pcaT*; however, these genes form an operon based on RT-PCR analysis with RNA isolated from strains grown in 4-hydroxybenzoate (unpublished).

**Suppl. Figure 6**. Genetic organization of the duplicated *cat* genes located in two different chromosomal regions. One of the *cat* clusters includes the *catR* gene that probably controls the expression of the two *catABC* operons based on high sequence conservation of the promoter region in the two *catB* genes.

**Suppl. Figure 7.** Genes and enzymes for the utilization of methionine as an S source by DOT-T1E. The gene products involved in the catabolism of methionine were identified based on BLAST analyses.

**Suppl. Figure 8**. Set of biotransformation reactions to achieve the synthesis of adipate / 3-oxoadipyl-CoA based on gene content in DOT-T1E strain. Multiple steps are represented by X^1^, X^2^ and X^3^. The loci are:

X^1^.- T1E-0314, T1E-0333, T1E-0690, T1E-0898, T1E-1004, T1E-1466, T1E1534, T1E-2226, T1E-2233, T1E-2397, T1E-3843, T1E-3853, T1E-3860, T1E-4416, T1E-5253, T1E-5478; X^2^.- T1E-0103, T1E-1864, T1E-1899, T1E-2485, T1E-2953, T1E-3631, T1E-4716, T1E-4861, T1E-4862; X^3^.- T1E-0369,T1E-1262, T1E-4182, T1E-4445

**References**

Del Castillo, T., Ramos, J.L., Rodríguez-Herva, J.J., Fuhrer, T., Sauer, U., and Duque, E. (2007) Convergent peripheral pathways catalyze initial glucose catabolism in *Pseudomonas putida*: genomic and flux analysis. *J Bacteriol* **189**: 5142-5152.

Jiménez, J.L., Miñambres, B., García, J.L., and Díaz, E. (2002) Genomic analysis of the aromatic catabolic pathways from *Pseudomonas putid*a KT2440. *Environ Microbiol* **4**: 824-841.

Nelson, K.E., Weinel, C., Paulsen, I.T., Dodson, R.J., Hilbert, H., Martins dos Santos V.A., Fouts, D.E., Gill, S.R., *et al*., (2002) Complete genome sequence and comparative analysis of the metabolically versatile *Pseudomonas putida* KT2440. *Environ Microbiol* **4**: 799-808.

Revelles, O., Wittich, R.-M., and Ramos, J.L. (2007) Identification of the initial steps in *D*-lysine catabolism in *Pseudomonas putida*. *J Bacteriol* **189**:2787-2792.

Suppl. Table 1. *P. putida* DOT-T1E enzymes of according to 4 digits EC classification

Suppl. Table 2. C, N and S sources identified *in silico* and used by *P. putida* DOT-T1E confirmed experimentally. For experimental details, see Daniels *et al*. J. Bacteriol. 192:22169-2181 (2010).

**Carbon source**

**Nitrogen source**

**Sulfur source**

Suppl. Table 3 Dioxygenases encoded by the *P. putida* DOT-T1E genome.

Suppl. Table 4. Monooxygenases encoded by the *P. putida* DOT-T1E genome.
